# CD68^+^ Macrophage Infiltration Associates With Poor Outcome of HPV Negative Oral Squamous Carcinoma Patients Receiving Radiation: Poly(I:C) Enhances Radiosensitivity of CAL-27 Cells but Promotes Macrophage Recruitment Through HMGB1

**DOI:** 10.3389/fonc.2021.740622

**Published:** 2021-09-09

**Authors:** Dan Ai, Yu Dou, Zhaodi Nan, Ketao Wang, Huayang Wang, Lin Zhang, Zuoqing Dong, Jintang Sun, Chao Ma, Wanye Tan, Wenjuan Gao, Jia Liu, Lei Zhao, Shaohua Liu, Bingfeng Song, Qianqian Shao, Xun Qu

**Affiliations:** ^1^Laboratory of Basic Medical Sciences, Qilu Hospital, Cheeloo College of Medicine, Shandong University, Jinan, China; ^2^Laboratory of Basic Medical Sciences, Qilu Hospital of Shandong University, Jinan, China; ^3^School and Hospital of Stomatology, Cheelo College of Medicine, Shandong University, Jinan, China; ^4^Department of Oral and Maxillofacial Surgery, Qilu Hospital of Shandong University & Institute of Stomatology, Shandong University, Jinan, China; ^5^Department of Clinical Laboratory Medicine, Qilu Hospital of Shandong University, Jinan, China

**Keywords:** oral squamous cell carcinoma, human papillomavirus, radiosensitivity, poly(I:C), HMGB1

## Abstract

Patients with human papillomavirus (HPV) negative oral squamous cell carcinoma (OSCC) generally have poor clinical outcomes and worse responses to radiotherapy. It is urgent to explore the underlining mechanisms of the distinct prognoses between HPV negative and HPV positive OSCC and to develop effective therapy strategy to increase the survival rate of HPV negative OSCC patients. We conducted a retrospective cohort of 99 resected OSCC patients to evaluate the prognosis of HPV negative and HPV positive OSCC patients receiving radiation or not. We further addressed the association of CD68^+^ macrophage infiltration with HPV status and the effects on survival of OSCC patients. We also used the TCGA-OSCC cohort for further verification. Based on the cohort study, we applied a synthetic dsRNA polymer, polyriboinosinic-polyribocytidylic acid (poly(I:C)), on CAL-27 (HPV negative OSCC cells). We co-cultured its condition medium with THP-1 derived macrophage and examined the cytokines and macrophage migration. We found that high CD68^+^ macrophage infiltration associated with poor overall survival in HPV negative OSCC patients receiving radiation. *In vitro*, poly(I:C) could induce apoptosis and enhance the radiosensitivity, but increase macrophage recruitment. Targeting HMGB1 could inhibit IL-6 induction and macrophage recruitment. Our findings indicated that CD68^+^ macrophage might play an important role in the outcomes of HPV negative OSCC patients receiving radiation. Our findings also suggested that radiation combined poly(I:C) might be a potential therapy strategy to increase the radiation response and prognosis of HPV negative OSCC. Notably, HMGB1 should be targeted to inhibit macrophage recruitment and enhance overall therapy effects.

## Introduction

Oral cancer, the most common head and neck cancer (HNC), accounts for more than 300,000 new cases of and 170,000 deaths occur worldwide per year (1). Oral squamous cell carcinoma (OSCC) comprises approximately 90% of these cases with a 5-year survival rate of 40–60% (2). Patients with human papillomavirus (HPV) negative OSCC generally have a poor prognosis and worse response to radiotherapy or chemoradiotherapy (3–5). HPV negative and positive OSCC exhibit distinct clinic-pathological features and heterogeneous microenvironments; however, the factors responsible for the distinct responses and prognoses remained obscure (6). Therefore, it is urgent to explore the underlining mechanisms of the distinct prognoses between HPV negative and positive OSCC, so as to optimize therapy strategy of HPV negative OSCC patients and increase overall survival rate.

Radiotherapy affects the tumor microenvironment, which in turn affects radiation-induced anticancer efficacy (7). Recent studies showed that enriched inflammatory lymphocyte infiltration in tumor microenvironment associated with HPV positive HNC and favorable prognosis (8–11). Macrophages are crucial drivers of tumor-promoting inflammation (12, 13). Macrophage polarization has also been reported to increase radiosensitivity in HPV positive HNC (14). So far, however, it is not clear whether macrophage infiltration associates with response to radiotherapy and survival of HPV negative and HPV positive OSCC. On the other hand, Hanoteau et al. reported that immune modulation of tumor microenvironment enhanced response to chemoradiotherapy of HNC (15). Sato-Kaneko et al. found that adjuvant toll like receptor (TLR) agonists could enhance tumor suppression and metastasis prevention of checkpoint inhibitors in HNC (16). Therefore, we hypothesized that TLR agonists might modulate tumor microenvironment and enhance the radiosensitivity of HPV negative OSCC, the majority population of OSCC.

We conducted a retrospective cohort of 99 resected OSCC patients and validated the findings using TCGA-OSCC cohort. We evaluated the association of CD68^+^ macrophage infiltration with HPV status and overall and disease-free survival of OSCC patients receiving or not receiving post operation radiation. Based on the findings of the cohort study, *in vitro*, we applied a synthetic dsRNA polymer, polyriboinosinic-polyribocytidylic acid [poly(I:C)], as a TLR agonist on CAL-27 (HPV negative OSCC cells). We assessed the apoptosis and proliferation of CAL-27 in response to poly(I:C) or combined with radiation. We co-cultured its condition medium with THP-1 derived macrophage and examined the induced cytokine profile and macrophage migration. We also addressed the role of a radiation injury associated molecule, High Mobility Group Box 1 (HMGB1), in above effects on macrophage.

## Materials and Methods

### Patient and Study Design

The specimens were obtained from 99 primary OSCC patients admitted in Qilu Hospital of Shandong University between 2006 and 2015. All patients received surgical resections without preoperative chemotherapy or radiotherapy. The ethical approval of this study was obtained from the Ethics Committee of Qilu Hospital of Shandong University. The patients were subject to radiotherapy according to TNM stage, tumor differentiation, and the patients’ intentions. The patients were followed up until May 2019 (median: 60 months). The baseline clinic-pathological characteristics were shown in **Supplementary Table S1**.

**Figure 1:**
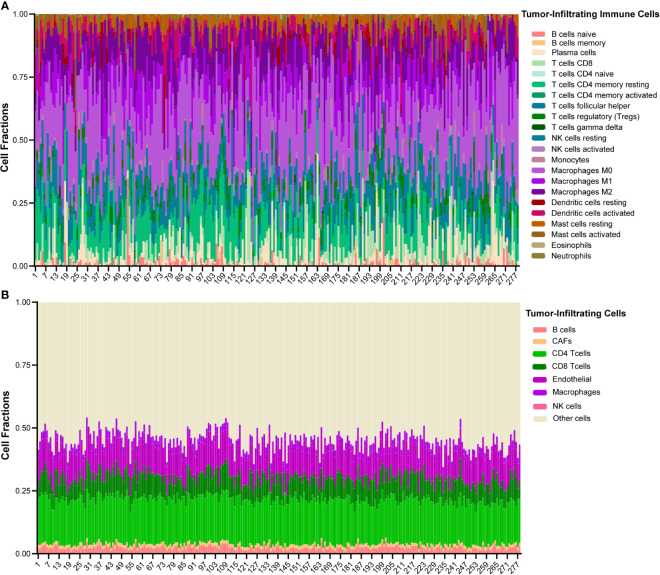
The landscape of immune infiltration of 278 OSCC patients in TCGA cohort. **(A)** Heatmap of 22 types of tumor-infiltrating immune cells deconvolved using CIBERSORT in OSCC. **(B)** Heatmap of 7 types of tumor-infiltrating cells deconvolved using EPIC in OSCC.

The Cancer Genome Atlas (TCGA)-HNSCC cohort was used for validation. The clinical characteristics of TCGA-HNSCC cohort were obtained from the Genomic Data Commons (GDC, https://portal.gdc.cancer.gov/). Gene expression data of TCGA-HNSCC RNA-sequencing (RNA-seq) dataset was obtained from UCSC Xena (https://xena.ucsc.edu/). HPV status was determined based on Cao et al. (17). Patients with HPV-supporting reads > 100 were defined as HPV positive. Updated follow-up information was used based on Liu et al. (18). Patients without HPV status or follow-up information were excluded. Patients with histories of malignancies and/or adjuvant therapies were also excluded. At last, a total of 278 OSCC patients were used for validation. The baseline clinic-pathological characteristics were shown in **Supplementary Table S2**. CIBERSORT, a deconvolution algorithm, was used to analyze the infiltration of macrophages in the tumor microenvironment (19) (https://cibersort.stanford.edu/index.php). Twenty-two human immune cell types were inferred. The landscape of immune infiltration is shown in **Figure 1A**. Another deconvolution tool, Estimating the Proportions of Immune and Cancer cells (EPIC), was also used to estimate the proportions of macrophages (20) (http://epic.gfellerlab.org). Seven human cell types were inferred by ERIC, and the landscape is shown in **Figure 1B**.

### Immunohistochemistry of p16 and CD68

P16 and CD68 were used to determine HPV status (4, 21) and macrophage infiltration (12, 22), respectively. Formalin-fixed paraffin-embedded tumor specimens were cut into 5-μm sections and processed for immunohistochemistry. Briefly, after incubation with a mouse anti-P16INK4a monoclonal antibody (1:50, 550834, BD Pharmingen, USA) or a mouse anti-CD68 (PG-M1) monoclonal antibody (ZM-0464, ZSGB-bio, China) at 4°C overnight, the sections were processed using biotin-streptavidin horseradish peroxidase detection system (SP-9000, SPlink Detection Kit, ZSGB-bio, Beijing, China). The slides were viewed under the Olympus IX81 microscope (Olympus, Japan), and the images were produced using DP Controller (Olympus, Japan).

P16 expression was evaluated based on staining intensity (0: no staining, 1: weak, 2: moderate, or 3: strong) and the proportion of stained tumor cells (0: 0–5%, 1: 6–25%, 2: 26–50%, 3: 51–75%, or 4: greater than 75%). P16 status was considered positive if staining intensity was strong (score 3) and the proportion of stained tumor cells was greater than 25% (score 2–4), or the staining intensity was moderate (score 2) and the proportion of stained tumor cells was greater than 75% (score 4). For CD68 evaluation, we counted the numbers of positively stained cells in five random fields (400×) in tumor nest of each specimen. The average number of CD68^+^ cells infiltrated in tumor nest per field was calculated. The level of CD68^+^ macrophages was determined according to its median value (12.67 per filed). All cases with number ≤ 12.67 per field were considered low, the number > 12.67 considered high. The evaluations of p16 and CD68 were performed by two pathologists and confirmed by another experienced pathologist.

### Cell Culture and Treatment

CAL-27 (HPV-negative human oral squamous cell line) and THP-1 (human monocytic cell line) were obtained from American Type Culture Collection (ATCC). All cells were cultured in phenol red-free Roswell Park Memorial Institute (RPMI) 1640 medium (HyClone, USA) supplemented with 10% charcoal stripped fetal bovine serum (HyClone, USA) at 37°C, 5% CO2.

CAL-27 were treated with 10 μg/ml poly(I:C) (TOCRIS, R&D, USA) or PBS for specific period of time (2 or 24 h). The cells were exposed with a serial of doses of radiation (0, 2, 6, or 8 Gy). Conditioned cells and conditioned medium (CM) were collected after culture for 24 h. Radiation was carried out using Varian 23EX 554 accelerator radiation platform in Department of Radiotherapy of Qilu Hospital of Shandong University. The required doses, 2, 6, and 8 Gy, were calculated according to 6MV X-ray PDD table of Varian 23EX 554 accelerator. The vertical irradiation field was 20 cm × 20 cm.

To generate THP-1-derived macrophages, THP-1 cells (1 × 10^6^ cells/well) were treated with 100 ng/ml phorbol myristate acetate (R&D, USA) for 6 h. For cytokine induction, the medium of THP-1-derived macrophages was replaced with 50% CAL-27 CM (or no CM control), which was the supernatants of CAL-27 treated with/without poly(I:C) for 24 h and/or 8 Gy radiation. The cells were continued to culture for 42 h before changing fresh medium and were further incubated for 24 h. IL-6 NAb neutralizing antibody or its isotype antibody (NAb/IsoAb, 10 μg/ml, R&D, USA), or HMGB1 NAb/IsoAb (1 μg/ml, Sigma, USA) was applied for the specific treatment groups as indicated.

### Cell Proliferation Assay

Cell proliferation was measured by CCK-8 assay (Bioss, China). CAL-27 cells were seeded in 96-well plates at a density of 2 × 10^3^ cells/200 μl. At the end of poly(I:C) and radiation treatments, the medium was replaced and CCK-8 solution (10 μg) was added to each well. At last, the optical density was measured at 450 nm with a microplate microscope after incubated in darkness for 2 h.

### Apoptosis Assay

Apoptosis was measured using Annexin V-FITC/PI apoptosis detection kit (BestBio, China). CAL-27 cells described above were collected and washed with cold PBS. The cells were then resuspended in 500 μl binding buffer containing 5 μl Annexin V-FITC and 5 μl propidium iodide and incubated for 15 min before analyzed for flow cytometry (NovoCyte, ACEA Biosciences, USA).

### Enzyme-Linked Immunosorbent Assay

The concentration of High Mobility Group Box 1 (HMGB1) in CAL-27 CM was determined by Human HMGB1 ELISA kit (Elabscience, China) according to the manufacturer’s instructions.

### Cytokine Assay

The supernatants of THP-1-derived macrophages after incubation with CAL-27 CM were collected and stored at -80°C after removal of the cell debris. Bio-Plex ProTM Human Th17 Cytokine Assay (Bio-rad, USA) was used to detect the levels of cytokines in above supernatants according to the manufacturer’s instructions.

### Migration Assay

The 24-well Transwell culture inserts (8 μm, BD Biosciences, USA) were used for cell migration assay according to the manufacturer’s instructions. THP-1-derived macrophages (1 × 10^5^) were resuspended in 100 μl of serum-free RPMI-1640 medium and seeded into the upper compartment of each well. RPMI-1640 medium (600 μl) with 10% FBS and 50% CAL-27 CM was added into the lower chamber of the plate. RPMI-1640 medium with 10% FBS but without CAL-27 CM was used as control. IL-6 NAb/IsoAb (10 μg/ml, R&D, USA) or HMGB1 NAb/IsoAb (1 μg/ml) was added into CAL-27 CM as indicated. After incubation at 37°C in 5% CO2 for 24 h, the migrated cells were fixed using 10% formalin and stained with eosin. Cell numbers of five random fields were counted, and images were taken.

### Statistical Analysis

Data were presented as mean ± standard deviation (SD) unless indicated. Student’s t test, Mann-Whitney U test, or ANOVA was used to determine the statistical significances as indicated. Chi-square test or Fisher’s exact test was used to determine the differences of clinicopathological characteristics between different groups. Kaplan-Meier analysis was used, and the log-rank test was used to discriminate the differences. Univariate and multivariate cox regressions were used to assess the association with overall or disease-free survival. Factors associated with cancer-specific survival with a *P* value lower than 0.1 and those shown to associate with cancer outcomes were further tested in multivariate cox regression. A two-tailed *P* value less than 0.05 was considered as statistical significance. For statistical analyses of cohort studies, IBM SPSS software version 25.0 (SPSS Inc., USA) was used. For statistical analyses of *in vitro* experiments, Graphpad Prism 8 software (La Jolla, USA) was used. All statistical graphs were generated using Graphpad Prism 8 software.

## Results

### Poor Overall Survival of HPV Negative OSCC Patients Receiving Radiation

**Figure 2:**
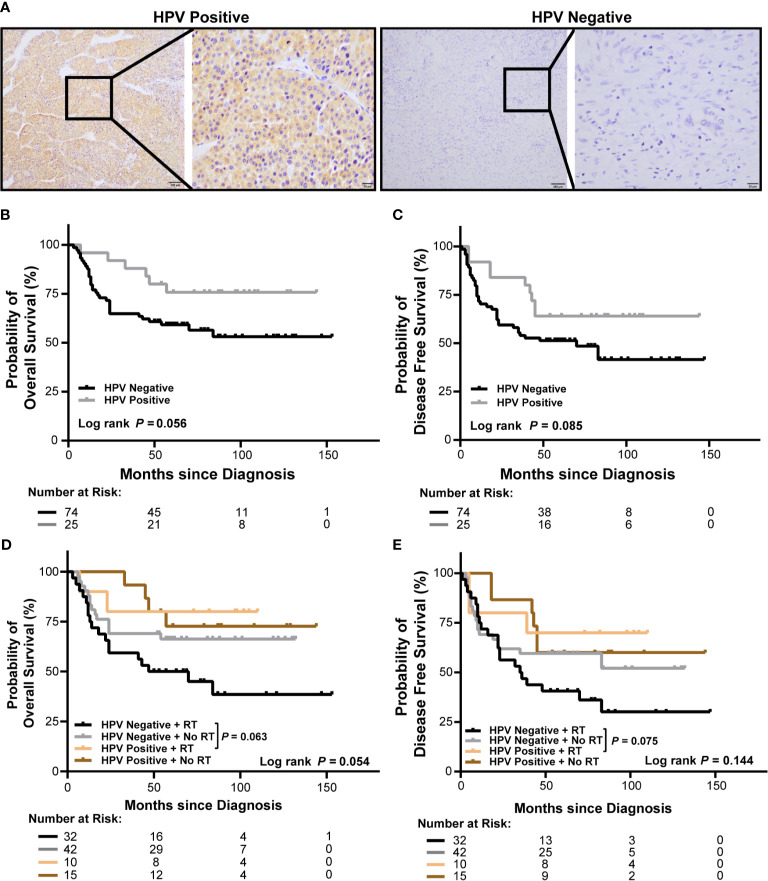
Association of HPV status and radiation with survival of OSCC patients in our cohort. **(A)** Representative immunohistochemical images of p16 positive and negative OSCC (left: 100×, right: 400×). **(B, C)** Kaplan-Meier curves show overall survival **(B)** and disease-free survival **(C)** of HPV negative and HPV positive OSCC patients. **(D, E)** Kaplan-Meier curves show overall survival **(D)** and disease-free survival **(E)** of HPV negative and HPV positive OSCC patients receiving radiation or no radiation. Log-rank test and/or pair wised comparison was used for significance. RT, radiation.

To analyze HPV status in OSCC, tumor specimens from 99 OSCC patients in our cohort were stained for p16 (**Figure 2A**), a marker of HPV. To investigate the association of HPV status with the outcomes of OSCC patients, we analyzed the survivals between HPV negative and HPV positive OSCC patients and between those received or not received radiation in our cohort. We observed a poor overall survival (OS) and a poor disease-free survival (DFS) in HPV negative patients with OSCC, however, the differences were not significant (*P* = 0.056 and *P* = 0.085, respectively, **Figures 2B, C**). Accordingly, there was no significant association between HPV status and OS or DFS in patients with OSCC in univariate or multivariate cox regression (hazard ratio: 0.439, 95% confidence interval: 0.183–1.053, *P* = 0.065 for OS, hazard ratio: 0.536, 95% confidence interval: 0.259–1.110, *P* = 0.093 for DFS, **Supplementary Tables S3, S4**). Furthermore, radiation treated OSCC patients with HPV negative status showed worse OS and DFS compared to those with HPV positive status; however, the differences were not significant either (*P* = 0.063 and *P* = 0.075, respectively, **Figures 2D, E**).

We further validated the findings in TCGA-OSCC cohort. OSCC patients (278) were selected from TCGA-HNSCC cohort as described in Materials and Method section. Trends of poor OS and DFS in HPV negative patients with OSCC were observed, however, the differences were not significant (*P* = 0.169 and *P* = 0.288, respectively, **Supplementary Figure S1A, B**). Accordingly, there was no association between HPV status and OS or DFS in patients with OSCC in univariate or multivariate cox regression (hazard ratio: 0.674, 95% confidence interval: 0.328–1.387, *P* = 0.284 for OS, hazard ratio: 0.623, 95% confidence interval: 0.327–1.188, *P* = 0.151 for DFS, **Supplementary Tables S5, S6**) in TCGA-OSCC cohort. In addition, radiation treated OSCC patients with HPV negative status showed a trend of worse OS compared to those with HPV positive status, but without statistical significance (*P* = 0.085, **Supplementary Figure S1C**).

The correlations between HPV status and clinic-pathological characteristics were also analyzed. No significant correlation was found in our cohort (**Supplementary Table S1**). More HPV negative patients were found in late stage OSCC patients in TCGA cohort (**Supplementary Table S2**).

### Intratumor CD68^+^ Macrophage Infiltration Is Not Correlated HPV Status or Outcomes of OSCC Patients

**Figure 3:**
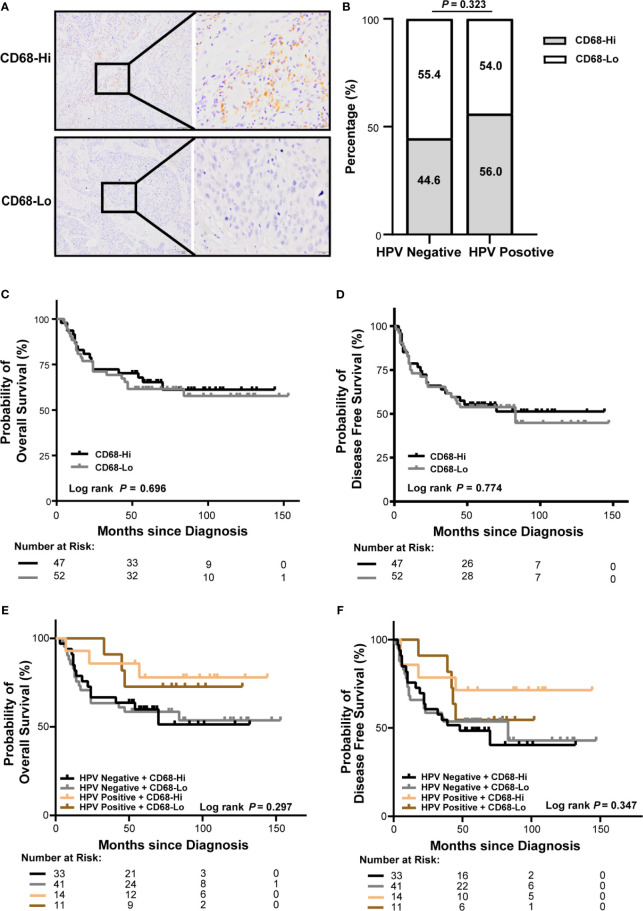
Association of CD68^+^ macrophage infiltration and HPV status with survival of OSCC patients in our cohort. **(A)** Representative immunohistochemical images of high or low level of CD68^+^ macrophages in OSCC (left: 100×, right: 400×). **(B)** Proportion of high or low level of CD68^+^ macrophages in patients with HPV negative and HPV positive OSCC. **(C, D)** Kaplan-Meier curves exhibit overall survival **(C)** and disease-free survival **(D)** in high or low level of CD68^+^ macrophage infiltrated OSCC patients. **(E, F)** Kaplan-Meier curves exhibit overall survival **(E)** and disease-free survival **(F)** in high or low level of CD68^+^ macrophage infiltrated OSCC patients with HPV negative and HPV positive status. Log-rank test and/or pair wised comparison was used for significance. CD68-Hi, CD68-High; CD68-Lo, CD68-Low; RT, radiation.

To analyze macrophage infiltration in OSCC, tumor specimens from 99 OSCC patients in our cohort were stained for CD68, a marker of human macrophages. As shown in **Figure 3A**, CD68^+^ cells present throughout the tumor core. Intratumor infiltration of CD68^+^ macrophages were evenly distributed in patients with HPV negative and HPV positive OSCC (**Figure 3B**). There was no association of CD68^+^ macrophage infiltration with overall or disease-free survival using log-rank test (**Figures 3C, D**) or univariate or multivariate cox regression (**Supplementary Tables S3, S4**). The association was not different either considering HPV status (**Figures 3E, F**).

We validated the findings in TCGA-OSCC cohort. Accordingly, macrophage infiltration deconvolved using CIBERSOFT was not correlated with HPV status (**Supplementary Figure S2A**). There was no association of macrophage infiltration with overall or disease-free survival (**Supplementary Figures S2B, C** and **Supplementary Tables S5, S6**). The association was not different either considering HPV status (**Supplementary Figures S2D, E**). Similar results were also observed in macrophage infiltration deconvolved using EPIC (**Supplementary Figure S3**).

Notably, we found that CD68^+^ macrophage infiltration was significantly correlated with differentiation in patients with HPV negative OSCC (*P* = 0.014), but not in those with HPV positive OSCC (*P* = 0.072, **Table 1**) in our cohort. In TCGA-OSCC cohort, we found that macrophage infiltration deconvolved using CIBERSOFT was correlated with the histological grade (*P* = 0.012, **Table 2**), especially in patients with HPV negative OSCC (*P* = 0.011, **Table 3**). In addition, we found that macrophage infiltration deconvolved using EPIC was also correlated with the histological grade in patients with HPV negative OSCC (*P* = 0.022, **Table 4**).

**Table 1:** Correlation of intratumor CD68^+^ macrophage infiltration with clinic-pathological characteristics of HPV positive and negative OSCC patients in our cohort.

Clinic-pathological characteristics	Total 25 *N* (%)	HPV positive patients		*P*^a^ value	Total 74 *N* (%)	HPV negative patients		*P*^b^ value
		CD68-high	CD68-low			CD68-high	CD68-low	
		14 (56.0%)	11 (44.0%)			33 (44.6%)	41 (55.4%)	
		*N* (%)	*N* (%)			*N* (%)	*N* (%)	
Age				0.697				0.065
≤59 years	15 (60.0)	9 (64.3)	6 (54.5)		36 (48.6)	20 (60.6)	16 (39.0)	
>59 years	10 (40.0)	5 (35.7)	5 (46.5)		38 (51.4)	13 (39.4)	25 (61.0)	
Gender				0.241				0.080
Male	15 (60.0)	10 (71.4)	5 (46.5)		41 (55.4)	22 (66.7)	19 (46.3)	
Female	10 (40.0)	4 (28.6)	6 (54.5)		33 (44.6)	11 (33.3)	22 (53.7)	
Smoking status				1.000				0.609
Smoking	12 (48.0)	7 (50.0)	5 (46.5)		29 (39.2)	14 (42.4)	15 (36.6)	
Non-smoking	13 (52.0)	7 (50.0)	6 (54.5)		45 (60.8)	19 (57.6)	26 (63.4)	
Drinking status				0.414				0.239
Drinking	10 (40.0)	7 (50.0)	3 (27.3)		26 (35.1)	14 (42.4)	12 (29.3)	
Non-drinking	15 (60.0)	7 (50.0)	8 (72.7)		48 (64.9)	19 (57.6)	29 (70.7)	
Tumor size				1.000				0.694
≤2 cm	14 (56.0)	8 (57.1)	6 (54.5)		34 (45.9)	16 (48.5)	18 (43.9)	
>2 cm	11 (44.0)	6 (42.9)	5 (46.5)		40 (54.1)	17 (51.5)	23 (56.1)	
Lymph node				1.000				0.093
Positive	8 (32.0)	5 (35.7)	3 (27.3)		28 (37.8)	9 (27.3)	19 (46.3)	
Negative	17 (68.0)	9 (64.3)	8 (72.7)		46 (62.2)	24 (72.7)	22 (53.7)	
TNM stage				1.000				0.419
Stage I–II	14 (56.0)	8 (57.1)	6 (54.5)		41 (55.4)	20 (60.6)	21 (51.2)	
Stage III–IV	11 (44.0)	6 (42.9)	5 (46.5)		33 (44.6)	13 (39.4)	20 (48.8)	
Differentiation				0.072				0.014
High	17 (68.0)	12 (86.0)	5 (46.5)		48 (64.9)	16 (48.5)	32 (78.0)	
Moderate	6 (24.0)	1 (7.0)	5 (46.5)		16 (21.6)	12 (36.4)	4 (9.8)	
Poor	2 (8.0)	1 (7.0)	1 (7.0)		10 (13.5)	5 (15.1)	5 (12.2)	
Radiotherapy				1.000				0.549
Yes	10 (40.0)	6 (42.9)	4 (36.4)		32 (43.2)	13 (39.4)	19 (46.3)	
No	15 (60.0)	8 (57.1)	7 (63.6)		42 (56.8)	20 (60.6)	22 (53.7)	

a: Fisher’s exact test. b: χ2 test.

**Table 2:** Correlation of macrophage infiltration deconvolved using CIBERSOFT and EPIC with clinic-pathological characteristics of OSCC patients in TCGA cohort.

Clinic-pathological characteristics	All patients278 (100%)*N (%)*	CIBERSORT		*P*^b^value	EPIC		*P*^b^value
		Macrophages-high	Macrophages-low		Macrophages-high	Macrophages-low	
		139 (50.0%)	139 (50.0%)		139 (50.0%)	139 (50.0%)	
		*N (%)*	*N* (%)		*N (%)*	*N* (%)	
Age				0.093			0.093
≤ 61 years	142 (51.1)	64 (46.0)	78 (56.1)		64 (46.0)	78 (56.1)	
>61 years	136 (48.9)	75 (54.0)	61 (43.9)		75 (54.0)	61 (43.9)	
Gender				0.305			0.073
Male	188 (67.6)	90 (64.7)	98 (70.5)		87 (62.6)	101 (72.7)	
Female	90 (32.4)	49 (35.3)	41 (29.5)		52 (37.4)	38 (27.3)	
Lymph node				0.102			0.421
Positive	143 (51.4)	69 (49.7)	74 (53.3)	0.936*	71 (51.1)	72 (51.8)	0.763*
Negative	111 (39.9)	53 (38.1)	58 (41.7)		53 (38.1)	58 (41.7)	
NA	24 (8.7)	17 (12.2)	7 (5.0)		15 (10.8)	9 (6.5)	
TNM stage				0.403^a^			0.494^a^
Stage I–II	73 (26.3)	41 (29.5)	32 (23.0)	0.204*	35 (25.2)	38 (27.3)	0.602*
Stage III–IV	198 (71.2)	94 (67.6)	104 (74.8)		102 (73.4)	96 (69.1)	
NA	7 (2.5)	4 (2.9)	3 (2.2)		2 (1.4)	5 (3.6)	
Histological grade				0.013^a^	0.013		0.057^a^
G1-G2	218 (78.4)	100 (72.0)	118 (84.9)	0.012*	102 (73.4)	116 (83.5)	0.052*
G3-G4	59 (21.2)	38 (27.3)	21 (15.1)		36 (25.9)	23 (16.5)	
NA	1 (0.4)	1 (0.7)	0 (0.0)		1 (0.7)	0 (0.0)	
Radiotherapy				1.000			0.054
Yes	154 (55.4)	77 (55.4)	77 (55.4)		85 (61.2)	69 (49.6)	
No	124 (44.6)	62 (44.6)	62 (44.6)		54 (38.8)	70 (50.4)	

a: Fisher’s exact test. b: χ2 test except for those marked with a. *Pairwise comparison without NA group.

**Table 3:** Correlation of macrophage infiltration deconvolved using CIBERSOFT with clinic-pathological characteristics of HPV positive and negative OSCC patients in TCGA cohort.

Clinic-pathological characteristics	Total33 (100%)*N (%)*	HPV positive patients		*P*^b^value	Total2 (100%)*N (%)*	HPV negative patients		*P*^b^value
		Macrophages-high	Macrophages-low		Total	Macrophages- high	Macrophages- low	
		17 (51.5%)	16 (48.5%)			122 (49.8%)	123 (50.2%)	
		*N* (%)	*N* (%)			*N* (%)	*N* (%)	
Age				0.080				0.308
≤ 61 years	19 (57.6)	7 (41.2)	12 (75.0)		123 (50.2)	57 (46.7)	66 (53.7)	
>61 years	14 (42.4)	10 (58.8)	4 (25.0)		122 (49.8)	65 (53.3)	58 (46.3)	
Gender				1.000				0.261
Male	27 (81.8)	14 (82.4)	13 (81.3)		161 (65.7)	76 (62.3)	85 (69.1)	
Female	6 (18.2)	3 (17.6)	3 (18.7)		84 (34.3)	46 (37.7)	38 (30.9)	
Lymph node				0.857				0.052
Positive	18 (54.5)	10 (58.8)	8 (50.0)	1.000*	125 (51.0)	59 (48.4)	66 (53.7)	0.974*
Negative	14 (42.4)	7 (41.2)	7 (43.8)		97 (39.6)	46 (37.7)	51 (41.4)	
NA	1 (3.1)	0 (0.0)	1 (6.2)		23 (9.4)	17 (13.9)	6 (4.9)	
TNM stage				1.000				0.505^a^
Stage I–II	11 (33.3)	6 (35.3)	5 (31.3)	1.000*	62 (25.3)	35 (28.7)	27 (22.0)	0.224*
Stage III–IV	17 (21.2)	8 (47.1)	9 (56.2)		181 (73.9)	86 (70.5)	95 (77.2)	
NA	5 (45.5)	3 (17.6)	2 (12.5)		2 (0.8)	1 (0.8)	1 (0.8)	
Histological grade				1.000				0.011^a^
G1–G2	28 (84.8)	14 (82.4)	14 (87.5)	1.000*	190 (77.6)	86 (70.5)	104 (84.6)	0.011*
G3–G4	5 (15.2)	3 (17.6)	2 (12.5)		54 (22.0)	35 (28.7)	19 (15.4)	
NA	0 (0.0)	0 (0.0)	0 (0.0)		1 (0.4)	1 (0.8)	0 (0.0)	
Radiotherapy				0.296				0.658
Yes	20 (60.6)	12 (70.6)	8 (50.0)		134 (54.7)	65 (53.3)	69 (56.1)	
No	13 (39.4)	5 (29.4)	8 (50.0)		111 (45.3)	57 (46.7)	54 (43.9)	

a: Fisher’s exact test. b: χ2 test except for those marked with a. *Pairwise comparison without NA group.

**Table 4:** Correlation of macrophage infiltration deconvolved using EPIC with clinic-pathological characteristics of HPV positive and negative OSCC patients in TCGA cohort.

Clinic-pathological characteristics	Total33 (100%)*N (%)*	HPV positive patients		*P*^b^value	Total245 (100%)*N (%)*	HPV negative patients		*P*^b^value
		Macrophages-high	Macrophages-low			Macrophages- high	Macrophages- low	
		18 (54.5%)	15 (45.5%)			121 (49.4%)	124 (50.6%)	
		*N* (%)	*N* (%)			*N* (%)	*N* (%)	
Age				1.000				0.085
≤ 61 years	19 (57.6)	10 (55.6)	9 (60.0)		123 (50.2)	54 (44.6)	69 (55.6)	
>61 years	14 (42.4)	8 (44.4)	6 (40.0)		122 (49.8)	67 (55.4)	55 (44.4)	
Gender				1.000				0.043
Male	27 (81.8)	15 (83.3)	12 (80.0)		161 (65.7)	72 (62.3)	89 (69.1)	
Female	6 (18.2)	3 (16.7)	3 (20.0)		84 (34.3)	49 (37.7)	35 (30.9)	
Lymph node				0.482				0.230
Positive	18 (54.5)	9 (50.0)	9 (60.0)	0.490*	125 (51.0)	62 (51.2)	63 (50.8)	0.531*
Negative	14 (42.4)	9 (50.0)	5 (33.3)		97 (39.6)	44 (36.4)	53 (42.7)	
NA	1 (3.1)	0 (0.0)	1 (6.7)		23 (9.4)	15 (12.4)	8 (6.5)	
TNM stage				0.132				0.942^a^
Stage I–II	11 (33.3)	5 (27.8)	6 (40.0)	0.248*	62 (25.3)	30 (24.8)	32 (25.8)	0.856*
Stage III–IV	17 (21.2)	12 (66.7)	5 (33.3)		181 (73.9)	90 (74.4)	91 (73.4)	
NA	5 (45.5)	1 (5.6)	4 (26.7)		2 (0.8)	1 (0.8)	1 (0.8)	
Histological grade				1.000				0.025^a^
G1–G2	28 (84.8)	13 (86.7)	15 (83.3)		190 (77.6)	86 (71.1)	104 (83.9)	0.022*
G3–G4	5 (15.2)	2 (13.3)	3 (16.7)		54 (22.0)	34 (28.1)	20 (16.1)	
NA	0 (0.0)	0 (0.0)	0 (0.0)		1 (0.4)	1 (0.8)	0 (0.0)	
Radiotherapy				0.039				0.216
Yes	20 (60.6)	14 (70.6)	6 (50.0)		134 (54.7)	71 (58.7)	63 (50.8)	
No	13 (39.4)	4 (29.4)	9 (50.0)		111 (45.3)	50 (41.3)	61 (49.2)	

a: Fisher’s exact test. b: χ2 test except for those marked with a. *Pairwise comparison without NA group.

### Intratumor CD68^+^ Macrophage Infiltration Associates With Poor Survival of HPV Negative OSCC Patients Receiving Radiation

**Figure 4:**
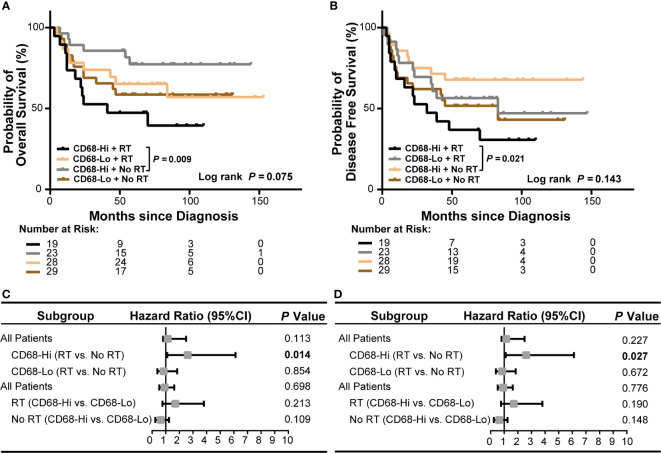
Association of CD68^+^ macrophage infilatration and radiation with survival of all OSCC patients in our cohort. **(A, B)** Kaplan-Meier curves show overall survival **(A)** and disease-free survival **(B)** in high or low level of CD68^+^ macrophage infiltrated OSCC patients receiving radiation or no radiation. Log-rank test and/or pair wised comparison was used for significance. **(C, D)** Forest plots illustrate hazard ratios of subgroup univariate cox regression of overall survival **(C)** and disease-free survival **(D)**. CD68-Hi, CD68-High; CD68-Lo, CD68-Low; RT, radiation.

**Figure 5:**
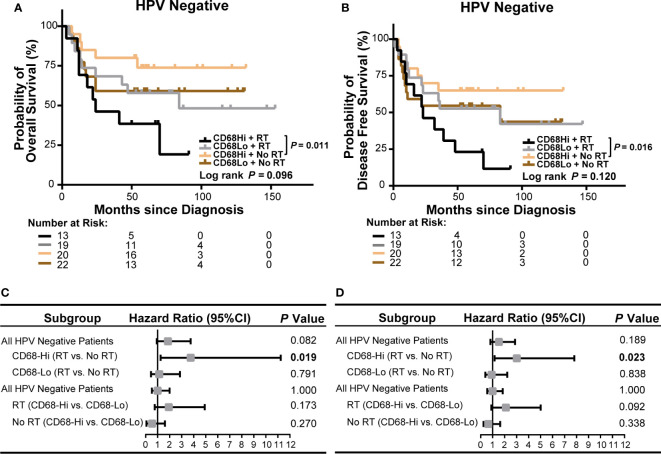
Association of CD68^+^ macrophage infilatration and radiation with survival of HPV negative OSCC patients in our cohort. **(A, B)** Kaplan-Meier curves exhibit overall survival **(A)** and disease-free survival **(B)** in high or low level of CD68^+^ macrophage infiltrated HPV negative OSCC patients receiving radiation or no radiation. Log-rank test and/or pair wised comparison was used for significance. **(C, D)** Forest plots illustrate hazard ratios of subgroup univariate cox regression of overall survival **(C)** and disease-free survival **(D)**. CD68-Hi, CD68-High; CD68-Lo, CD68-Low; RT, radiation.

We next evaluated the role of CD68^+^ macrophage infiltration in the outcomes of radiation treated OSCC patients in HPV negative and HPV positive subgroups in our cohort. We observed a poor OS and a poor DFS in high CD68^+^ macrophage infiltrated OSCC patients received radiation in all OSCC patients (*P* = 0.009 and *P* = 0.021, respectively, **Figure 4A, B**). In univariate cox regression model, high CD68^+^ macrophage infiltration was associated with poor OS and DFS of OSCC patients received radiation (hazard ratio: 3.492, 95% confidence interval: 1.287–9.476, *P* = 0.014 for OS, hazard ratio: 2.610, 95% confidence interval: 1.112–6.123, *P* = 0.027 for DFS, **Figure 4C, D** and **Supplementary Tables S7, S8**). Notably, similar results only appeared in the HPV negative OSCC subgroup (*P* = 0.011 and *P* = 0.016, respectively, **Figures 5A, B**). Accordingly, radiation was associated with poor OS and DFS of high CD68^+^ macrophage infiltrated OSCC patients in HPV negative subgroup in univariate cox regression model (hazard ratio: 3.746, 95% confidence interval: 1.248–11.244, *P* = 0.019 for OS, hazard ratio: 3.012, 95% confidence interval: 1.161–7.814, *P* = 0.023 for DFS, **Figures 5C, D** and **Supplementary Tables S9, S10**). However, the associations remained not statistically significant after adjusting for age, gender, tumor size, lymph node metastasis, and differentiation in multivariate cox regression model (**Supplementary Tables S9, S10**). In addition, there were no associations in HPV positive OSCC subgroup (**Supplementary Figure S4** and **Supplementary Tables S11** and **S12**) in our cohort. We also validated these findings in TCGA-OSCC cohort. However, no association of macrophage infiltration deconvolved using CIBERSOFT or EPIC and prognosis was observed in all OSCC patients (**Supplementary Figures S5, S6** and **Supplementary Tables S13–S16**), HPV negative OSCC subgroup (**Supplementary Figures S7, S8** and **Supplementary Tables S17–S20**), or HPV positive OSCC subgroup (data not shown). These results suggested that CD68^+^ macrophage might play an important role in the distinct prognoses of HPV negative and positive OSCC patients receiving radiation or not.

### Poly(I:C) Induces Apoptosis of CAL-27 and Enhances Its Radiosensitivity

**Figure 6:**
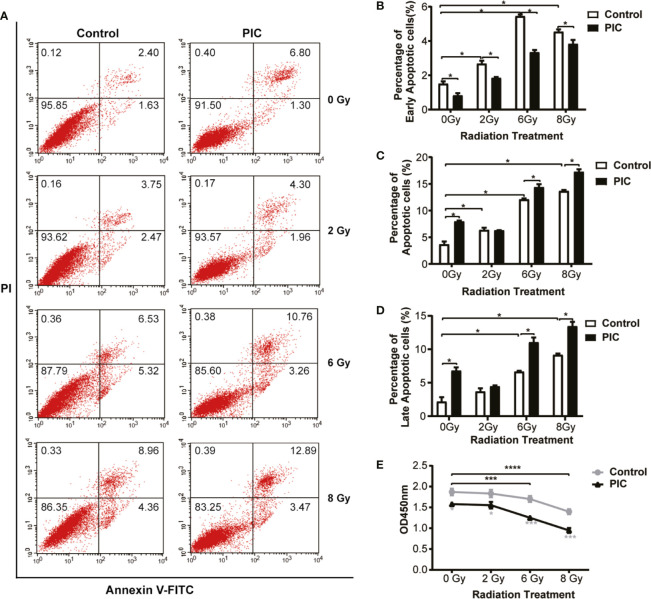
Poly(I:C) enhances radiation-induced apoptosis and inhibits proliferation of CAL-27. CAL-27 cells were treated with poly(I:C) or PBS for 24 h followed by radiation. **(A)** Representative plots illustrate apoptosis of CAL-27 according to annexin V and/or propidium iodide (PI) staining. **(B)** Bar plots indicate the quantifications of early apoptotic cells ((annexin V+/PI-) of the bottom right quadrant. **(C)** Bar plots indicate the quantifications of late apoptotic cells (annexin V+/PI+) of the top right quadrant. **(D)** Bar plots indicate the quantifications of total apoptotic cells (annexin V+) of the top right and bottom right quadrants. **(E)** The proliferation curve shows the proliferation of CAL-27 detected by CCK-8 assay. Student’s t-test was used for significance determination of flowcytometry (mean ± SD, n = 3). ANOVA was used for significance determination of CCK-8 assay (mean ± SD, n = 5). * indicates *P* < 0.05, *** indicates *P* < 0.001 and **** indicates *P* < 0.0001. PIC, poly (I:C).

In the above cohort studies, we observed trends of poor OS and DFS of radiation treated OSCC patients in HPV negative subgroup. To explore whether virus affects the radiosensitivity of HPV negative OSCC cells, *in vitro*, we applied a viral dsRNA mimic, poly(I:C), on CAL-27 cells. We performed apoptosis assay of flow cytometry of CAL-27 treated with poly(I:C) combined with radiation (**Figure 6A–D**). We demonstrated that the apoptotic rates of CAL-27 were significantly increased by increasing of radiation dose. On the other hand, poly(I:C) further increased the apoptosis rates of CAL-27 treated with radiation. Similarly, the proliferation of CAL-27 was also significantly decreased by increasing radiation dose (**Figure 6E**). Poly(I:C) had a further inhibitory effect on the proliferation of CAL-27 treated with radiation. Together, these results suggested that poly(I:C) not only induced apoptosis of CAL-27, but also enhanced the radiosensitivity of CAL-27.

### Poly(I:C) Alters Cytokine Induction and Recruitment of Macrophage Cocultured With Radiation Stimulated CAL-27

**Figure 7:**
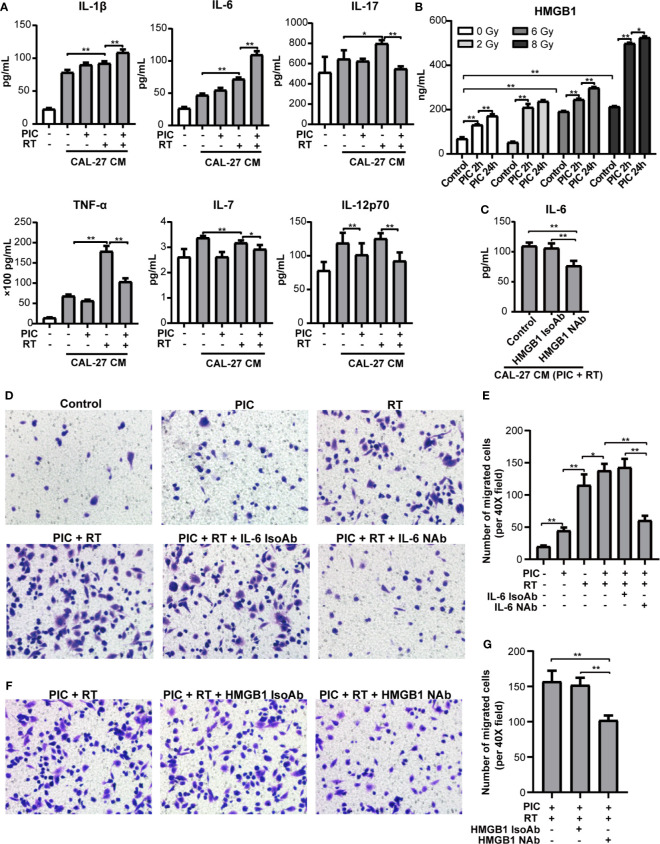
Poly(I:C) and radiation-stimulated CAL-27 alters cytokine secretion of macrophages and promotes macrophage recruitment through IL-6 and HMGB1. CAL-27 cells were treated with poly(I:C) or PBS for 24 h followed by 8 Gy or 0 Gy radiation. THP-1-derived macrophages were treated with above CAL-27 CM (or no CM control) and continued to culture for 42 h before incubation for 24 h with fresh medium. **(A)** The concentrations of IL-1β, IL-6, IL-17, TNF-α, IL-7, and IL-12p70 in the supernatants were detected by cytokine assay. ANOVA was used (mean ± SD, n = 5). **(B)** Bar plot shows HMGB1 concentrations in CAL-27 CM detected by ELISA. Mann-Whitney U-test was used (mean ± SD, n = 3). **(C)** Bar plot shows the concentration of IL-6 in the supernatants of THP-1-derived macrophages treated with CAL-27 CM (poly (I:C) 24 h combined 8 Gy radiation) or pretreated with HMGB1 neutralizing antibody or isotype antibody. **(D, E)** Representative images (×200) and quantifications of the migrated THP-1-derived macrophages treated with CAL-27 CM and IL-6 neutrolizing antibody (or isotype). **(F, G)** Representative images (×200) and quantifications of the migrated THP-1-derived macrophages treated with CAL-27 CM and HMGB1 neutrolizing antibody (or isotype). ANOVA was used (mean ± SD, n = 5). * indicates *P* < 0.05 and ** indicates *P* < 0.01. PIC, poly (I:C); CM, condition medium; RT, radiation. NAb, neutralizing antibody; IsoAb, isotype antibody.

In the above cohort studies, we found that radiation was associated with poor OS and DFS of high CD68^+^ macrophage infiltrated OSCC patients in HPV negative subgroup. To explore the effect of poly(I:C) and/or radiation treated CAL-27 on cytokine secretion of macrophage, we treated THP-1-derived macrophages with CAL-27 conditioned medium (CM) and evaluated the levels of cytokines using Th17 Cytokine Assay (**Figure 7A**). M1-type cytokines, IL-1β, IL-6, IL-17 and TNF-α of THP-1-derived macrophages were induced in response to radiation stimulated CAL-27 CM. Moreover, the inductions of IL-1β and IL-6 were also significantly increased in response to poly(I:C) combined radiation stimulated CAL-27 CM. The inductions of IL-17 and TNF-α were significantly decreased in response to poly(I:C) combined radiation stimulated CAL-27 CM. On the other hand, radiation-stimulated CAL-27 CM significantly inhibited the secretion of IL-7 in THP-1-derived macrophage, and the addition of poly(I:C) further inhibited the secretion of IL-7. Furthermore, poly(I:C) treated CAL-27 CM inhibited IL-12p70 secretion of THP-1-derived macrophages regardless of radiation treatment. These results addressed the importance of M1-type cytokine, IL-6, which was induced most significantly in response to both radiation and poly(I:C) stimulated CAL-27 CM.

Based on the evidences of HMGB1 in macrophage function and radiation damage, we further explored the induction of HMGB1 in poly(I:C) and/or radiation treated CAL-27 CM (**Figure 7B**) and its role in IL-6 induction (**Figure 7C**). HMGB1 induction of CAL-27 CM was significantly promoted in response to radiation in a dose dependent manner. On the other hand, poly(I:C) significantly promoted HMGB1 induction of CAL-27 in a time dependent manner regardless of radiation. Notably, poly(I:C) treatment for 24h combined 8Gy radiation showed the most significant induction of HMGB1. Furthermore, neutralizing HMGB1 significantly inhibited IL-6 induction of THP-1-derived macrophage in response to CAL-27 CM treated with poly(I:C) for 24h and 8Gy radiation.

To investigate the role of IL-6 in macrophage recruitment, we established an *in vitro* macrophage migration model using transwell chambers. We co-cultured THP-1-derived macrophages with CAL-27 CM treated with poly(I:C) and radiation. We found that poly(I:C) or radiation treated CAL-27 CM promoted the recruitment of THP-1-derived macrophages. The recruitment was significantly enhanced by combining poly(I:C) and radiation. However, the recruitment was depleted by neutralizing IL-6 (**Figures 7D, E**). These suggested the key role of IL-6 in macrophage recruitment by CAL-27 CM. Furthermore, neutralizing HMGB1 also significantly inhibited the recruitment of THP-1-derived macrophages by in response to CAL-27 CM (**Figures 7F, G**). These results suggested that poly(I:C) and radiation stimulated CAL-27 CM promoted macrophage recruitment could be inhibited by targeting HMGB1.

## Discussion

**Figure 8:**
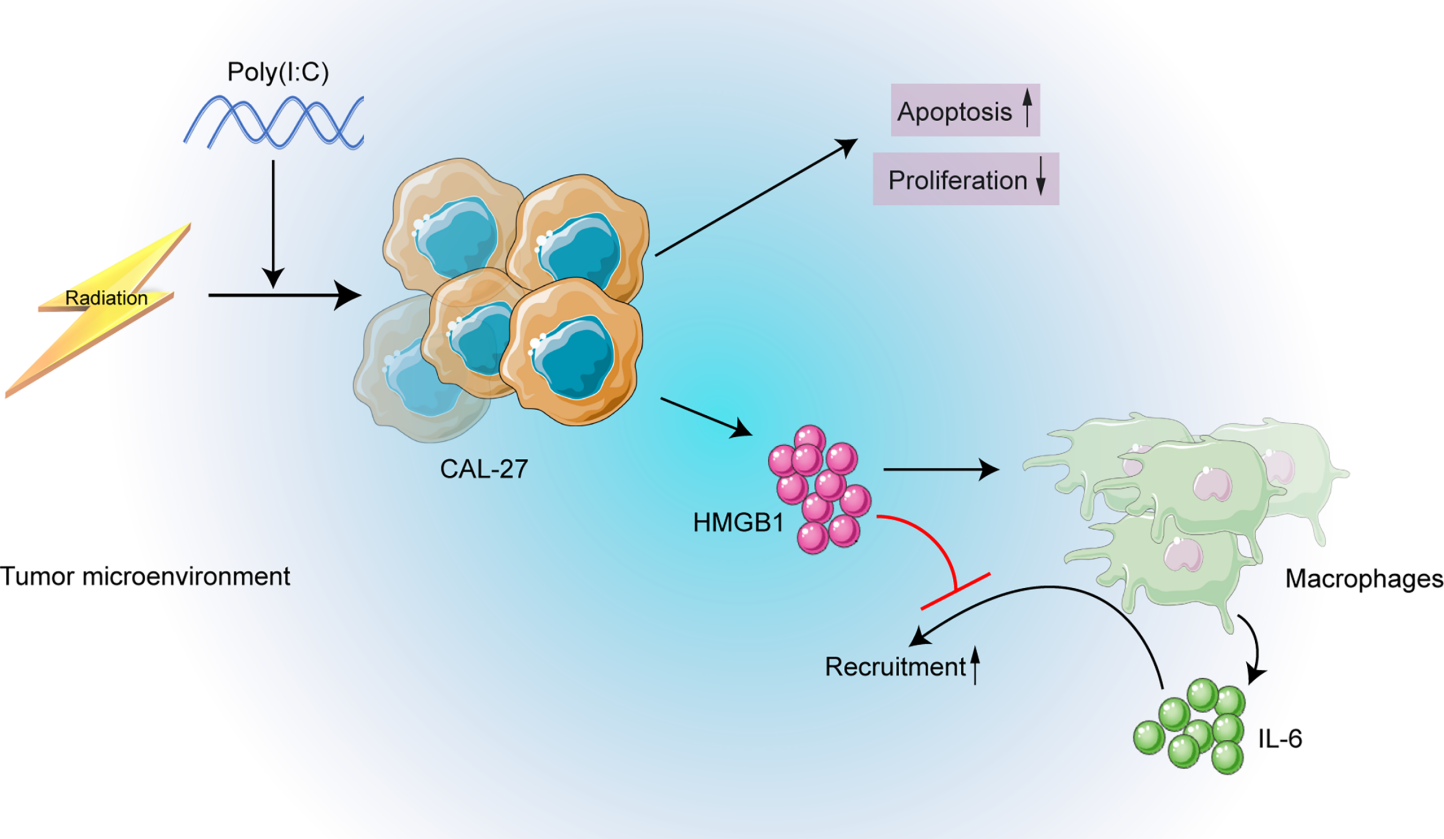
The schematic diagram depicting the strategy of targeting HMGB1 may enhance radiosensitivity of CAL-27 cells by inhibiting macrophage recruitment induced by poly(I:C).

The need to explore the underlining mechanisms of the distinct prognoses of HPV negative and positive OSCC is urgent for precise medicine. Lymphocyte infiltration of tumor microenvironment and its modulation have been shown associated with HPV positive HNC patients and their favorable prognosis and better therapy response (8–11, 15, 16). Nonetheless, the role of macrophage has been overlooked. We observed a poor OS and a poor DFS in high CD68^+^ macrophage infiltrated OSCC patients receiving radiation in HPV negative subgroup in our cohort. Based on the cohort results, we further conducted *in vitro* experiments, we found that poly(I:C) could not only induce apoptosis, but also enhanced the radiosensitivity of CAL-27. Furthermore, neutralizing IL-6 or HMGB1 could inhibit macrophage recruitment. A schematic diagram was made to depict the above mechanisms (**Figure 8**).

To explore the association of macrophage infiltration with HPV status and survival of OSCC patients, we conducted a retrospective cohort. In our study, we found that CD68^+^ macrophage infiltration was not associated with OS or DFS of OSCC patients. The association of CD68^+^ macrophages with survival of OSCC patients is controversial. Some studies showed that high CD68^+^ macrophage associated with poor survival of OSCC (23–27) or with favorable survival of OSCC (28). Consistent with our results, there are also studies demonstrated no association of CD68^+^ macrophage infiltration with prognosis of OSCC patients (29, 30). However, we observed a poor OS and a poor DFS in radiation treated OSCC patients with high CD68^+^ macrophage infiltration, especially in HPV negative subgroup, but not in HPV positive subgroup. However, the lack of association between macrophage infiltration and radiation response in HPV positive OSCC patients might also result from the limited number of HPV positive subgroup. These findings indicated that CD68^+^ macrophage might associate with poor radiation response and prognosis of HPV negative OSCC patients, and that CD68^+^ macrophage infiltration might need to be reduced before radiation therapy for HPV negative OSCC patients.

Since HPV negative OSCC patients receiving radiation obtained worse survival than HPV positive OSCC patients, we applied radiation and/or poly(I:C) to CAL-27 cells in an attempt to examine whether viral mimic could affect radiosensitivity of HPV negative OSCC. We found that poly(I:C) could induce apoptosis of CAL-27. This is consistent with previous studies of pancreatic cancer (31, 32), glioblastoma (33), and neuroblastoma (34). We also found that poly(I:C) could enhance the radiosensitivity of CAL-27. This is consistent with Mikulandra’s finding using poly(I:C) and cisplatin in HNSCC-derived cells (35) and Sato’s finding in lung adenocarcinoma (36). Together, these suggested that radiation combined with poly(I:C) could be potentially used for OSCC suppression.

On the other hand, we found that M1-type cytokines were induced in THP-1 derived macrophages in response to radiation and poly(I:C) stimulated CAL-27 CM. IL-6, the most significantly induced cytokine, played a key role in macrophage recruitment by CAL-27 CM. We also demonstrated that the process was dependent of HMGB1. It has been shown as a key damage-related molecular pattern to induce inflammation in response to radiation (37–39). However, HMGB1 has also been shown to promote hepatocellular carcinoma (40) and associate with radiation resistance of bladder cancer cells (41). Accordingly, in our study, neutralizing HMGB1 could inhibit IL-6 induction and macrophage recruitment. Taken together, our findings suggested that although poly(I:C) could enhance radiosensitivity of OSCC, neutralizing HMGB1 should also be used to inhibit macrophage recruitment promoted by poly(I:C) and radiation CAL-27 CM.

In summary, we demonstrated that CD68^+^ macrophage infiltration might associate with poor prognosis of HPV negative OSCC patients receiving radiation using our cohort. We found that treating CAL-27 with a viral mimic, poly(I:C), could induce apoptosis and enhance the radiosensitivity. Furthermore, HMGB1 should be targeted to inhibit macrophage recruitment and may enhance the overall therapy effects. Our findings may supply a potential therapy strategy to increase the radiation response and prognosis of HPV negative OSCC and provide new insights in understanding the molecular mechanisms.

## Data Availability Statement

The original contributions presented in the study are included in the article/**Supplementary Material**. Further inquiries can be directed to the corresponding authors.

## Ethics Statement

The studies involving human participants were reviewed and approved by The Ethics Committee of Qilu Hospital of Shandong University. Written informed consent for participation was not required for this study in accordance with the national legislation and the institutional requirements.

## Author Contributions

XQ and YD conceived and designed the project. KW, ZD, WT, and SL performed the surgeries and collected the clinical characteristics data. ZN, HW, LZhang and LZhao completed the follow-ups. DA, JS, and CM performed the immunohistochemistry and histological analyses. YD, DA, and ZN performed the survival analysis of the cohort. DA, HW, WG, JL, BS, and QS performed in vitro experiments and analyzed the data. YD and DA wrote the manuscript. All authors contributed to the article and approved the submitted version.

## Funding

This work was supported by the grants of XQ (81772879) and YD (81902770) from National Natural Science Foundation of China.

## Conflict of Interest

The authors declare that the research was conducted in the absence of any commercial or financial relationships that could be construed as a potential conflict of interest.

The handling editor and the reviewer DC declared a shared affiliation, though no other collaboration, with the authors at the time of the review.

## Publisher’s Note

All claims expressed in this article are solely those of the authors and do not necessarily represent those of their affiliated organizations, or those of the publisher, the editors and the reviewers. Any product that may be evaluated in this article, or claim that may be made by its manufacturer, is not guaranteed or endorsed by the publisher.
