# Supplementary material for: CD68+ Macrophage Infiltration Associates With Poor Outcome of HPV Negative Oral Squamous Carcinoma Patients Receiving Radiation: Poly(I:C) Enhances Radiosensitivity of CAL-27 Cells but Promotes Macrophage Recruitment Through HMGB1
Source: Front Oncol. 2021 Sep 9;11:740622. doi: 10.3389/fonc.2021.740622 (PMC8459684; doi:10.3389/fonc.2021.740622)
Supplement: Supplementary file 1 [file DataSheet_1.docx]

Supplementary Material

# Supplementary Figures and Tables

## Supplementary Figures


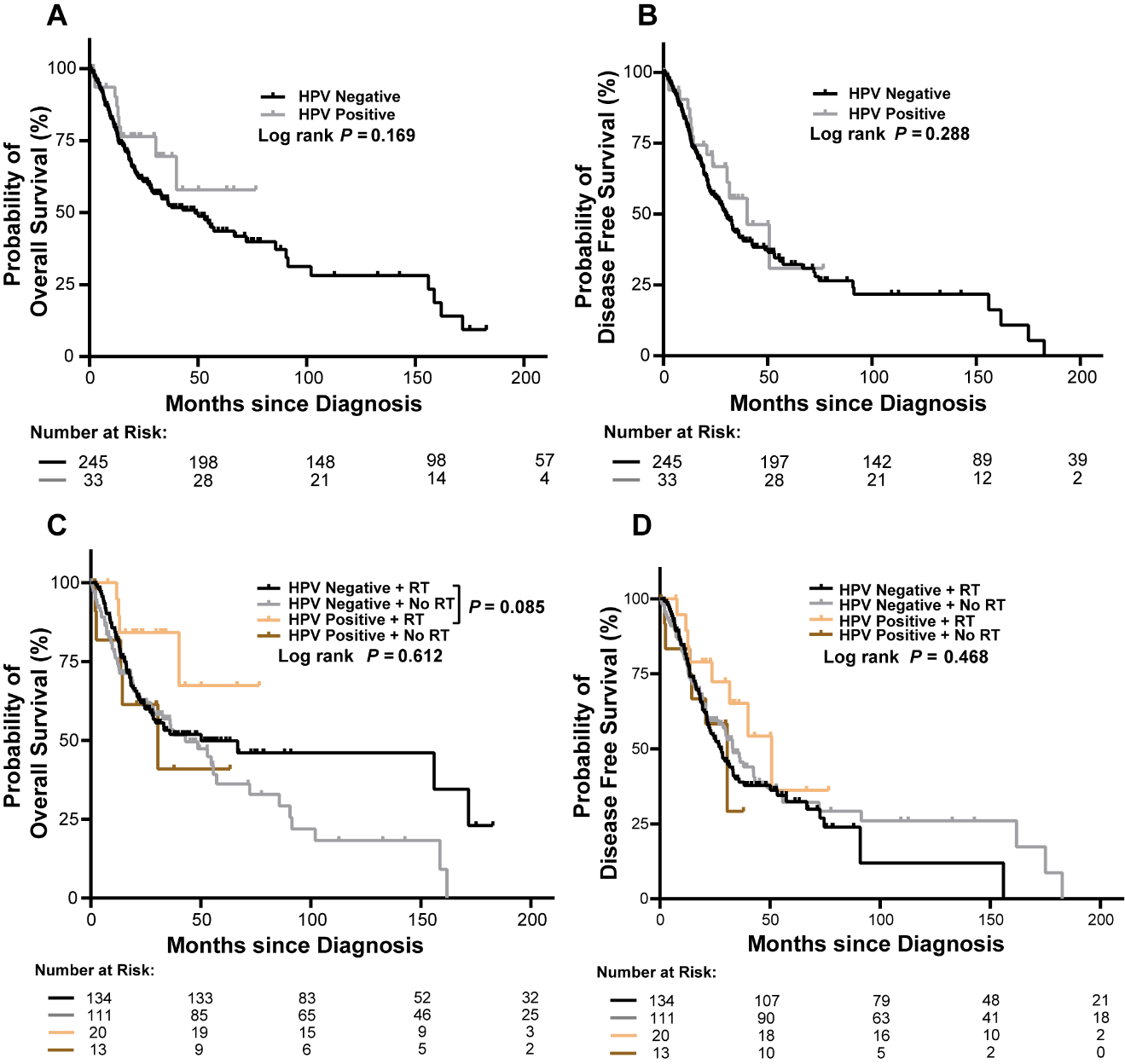


**Figure S1.** Association of HPV status and radiation with survival of OSCC patients in TCGA cohort. (**A** and **B**) Kaplan-Meier curves show overall survival (**A**) and disease-free survival (**B**) of HPV negative and HPV positive OSCC patients. (**C** and **D**) Kaplan-Meier curves show overall survival (**C**) and disease-free survival (**D**) of HPV negative and HPV positive OSCC patients receiving radiation or no radiation. Log-rank test and/or pair wised comparison was used for significance. RT: radiation. This figure corresponds to the findings of our cohort as shown in **Figure 2**.


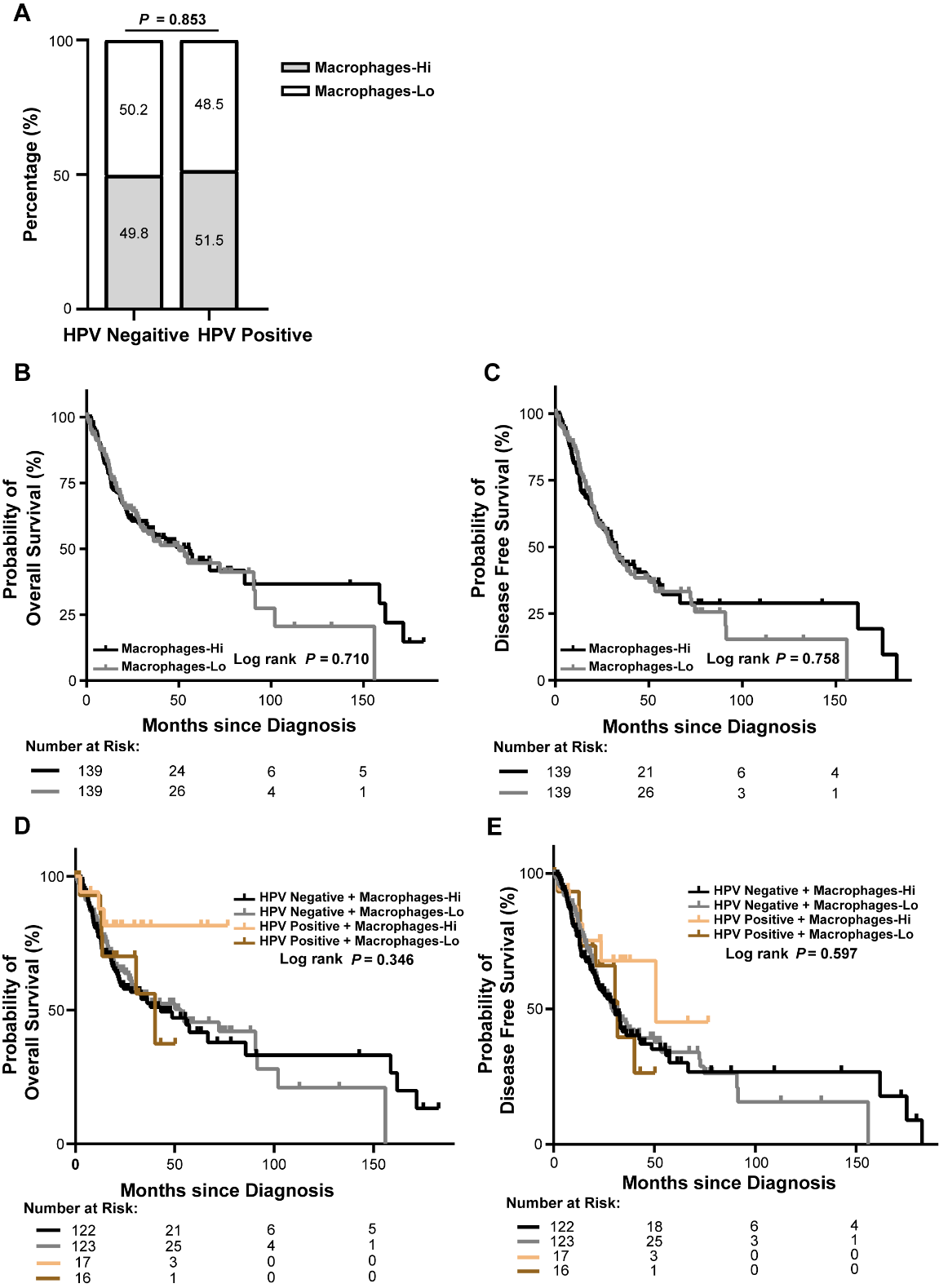


**Figure S2.** Association of macrophage infiltration deconvolved using CIBERSOFT and HPV status with survival of OSCC patients in TCGA cohort. (**A**) Proportion of high or low level of macrophage infiltration in patients with HPV negative and positive OSCC. (**B** and **C**) Kaplan-Meier curves show overall survival (**B**) and disease-free survival (**C**) of OSCC patients with high or low level of macrophage infiltration. (**D** and **E**) Kaplan-Meier curves exhibit overall survival (**D**) and disease-free survival (**E**) in high or low level of macrophage infiltration of OSCC patients with HPV negative and positive status. Log-rank test and/or pair wised comparison was used for significance. Macrophages-Hi: Macrophages-High, Macrophages-Lo: Macrophages-Low. This figure corresponds to the findings of our cohort as shown in **Figure 3**.


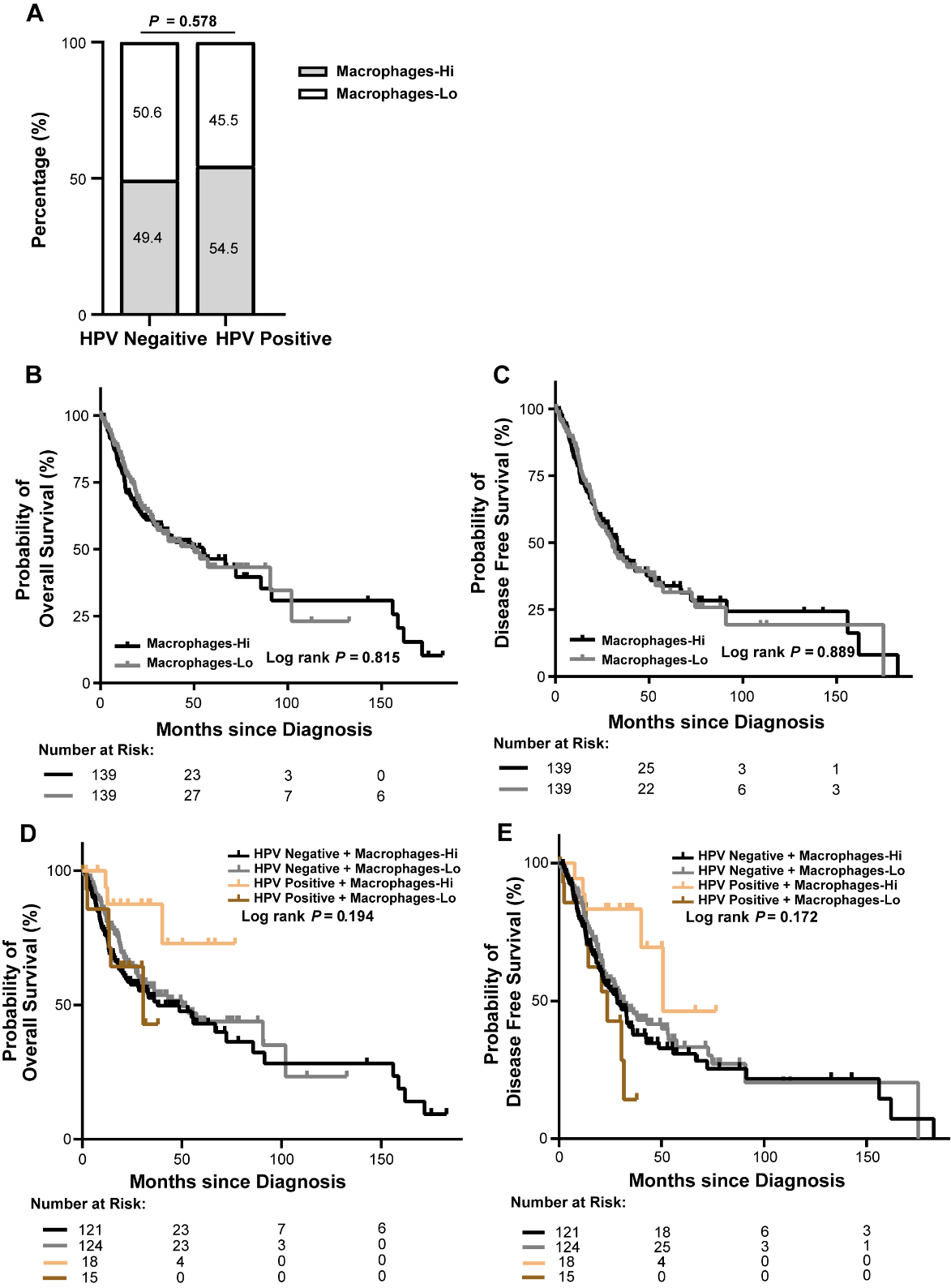


**Figure S3.** Association of macrophage infiltration deconvolved using EPIC and HPV status with survival of OSCC patients in TCGA cohort. (**A**) Proportion of high or low level of macrophage infiltration in patients with HPV negative and positive OSCC. (**B** and **C**) Kaplan-Meier curves show overall survival (**B**) and disease-free survival (**C**) of OSCC patients with high or low level of macrophage infiltration. (**D** and **E**) Kaplan-Meier curves exhibit overall survival (**D**) and disease-free survival (**E**) in high or low level of macrophage infiltration of OSCC patients with HPV negative and positive status. Log-rank test and/or pair wised comparison was used for significance. Macrophages-Hi: Macrophages-High, Macrophages-Lo: Macrophages-Low. This figure corresponds to the findings of our cohort as shown in **Figure 3**.

**
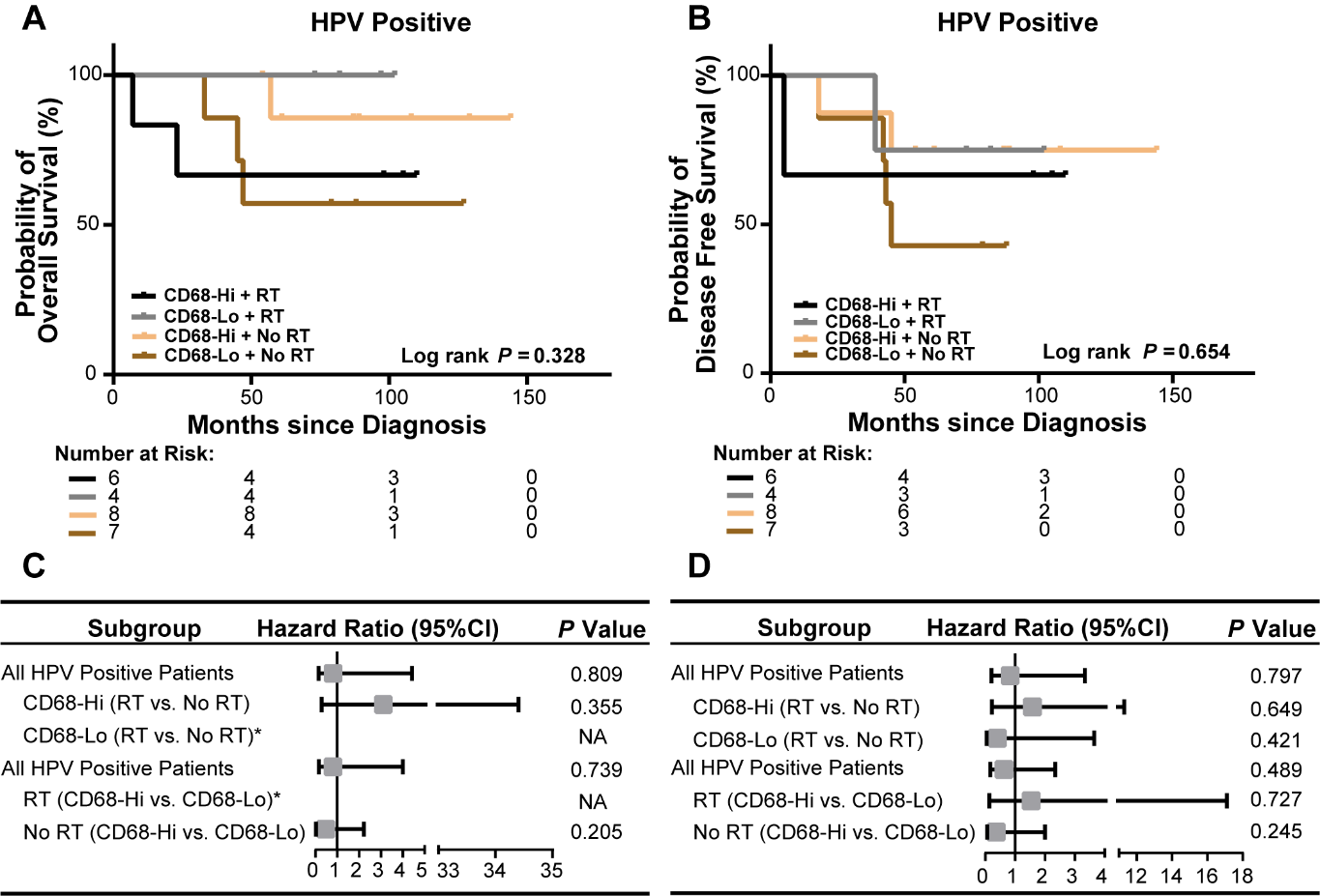
**

**Figure S4.** Association of CD68^+^ macrophage infilatration and radiation with survival of HPV positive OSCC patients in our cohort. (**A** and **B**) Kaplan-Meier curves show overall survival (**A**) and disease-free survival (**B**) in high or low level of CD68^+^ macrophage infiltrated HPV positive OSCC patients receiving radiation or no radiation. Log-rank test and/or pair wised comparison was used for significance. (**C** and **D**) Forest plots illustrate hazard ratios of subgroup univariate Cox regression of overall survival (**C**) and disease-free survival (**D**). CD68-Hi: CD68-High, CD68-Lo: CD68-Low, RT: radiation. NA and *: Cox regression is not applicable for marked subgroups.


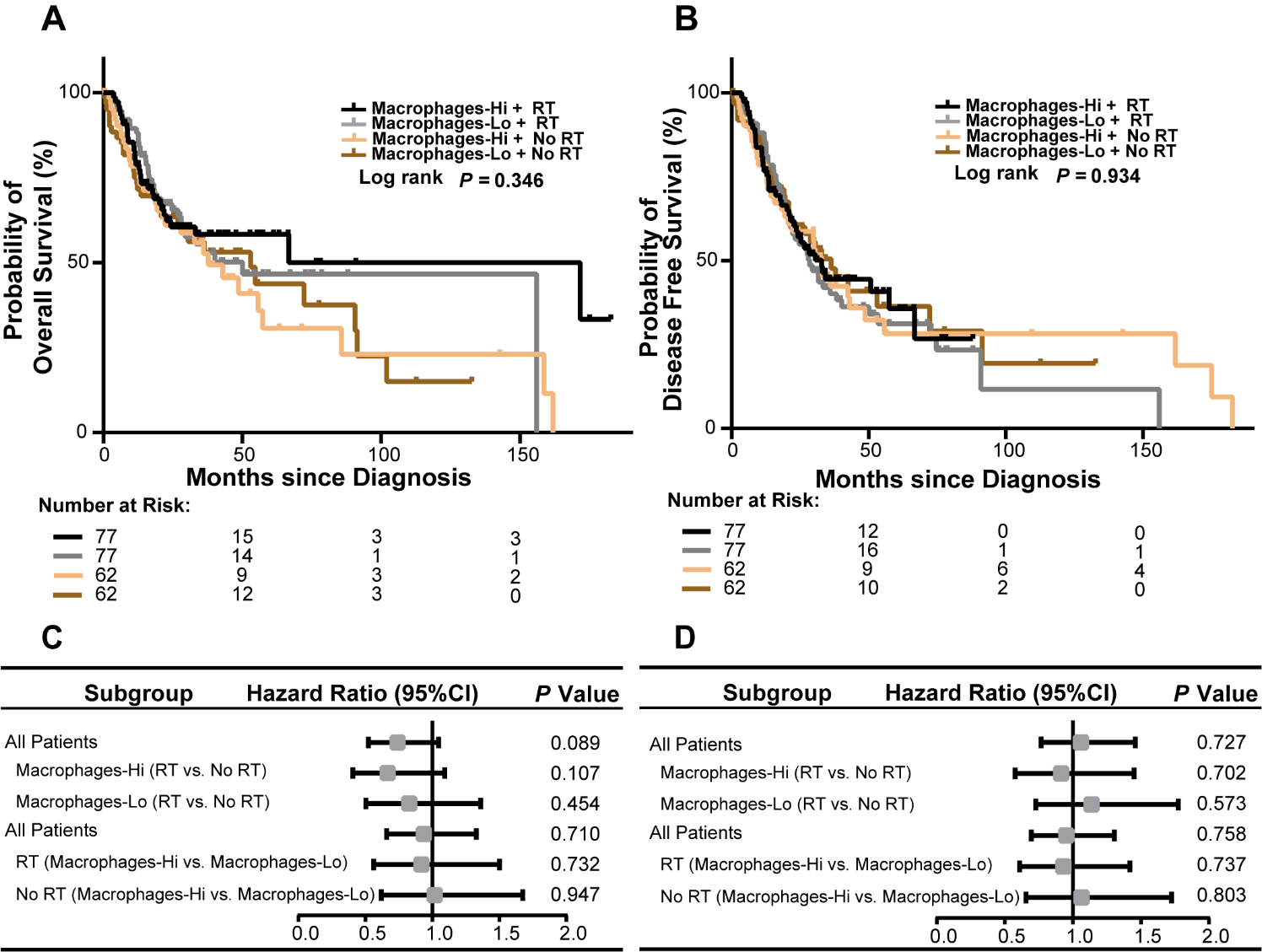


**Figure S5.** Association of macrophage infiltration deconvolved using CIBERSOFT and radiation with survival of all OSCC patients in TCGA cohort. (**A** and **B**) Kaplan-Meier curves show overall survival (**A**) and disease disease-free survival (**B**) in high or low macrophage infiltrated OSCC patients receiving radiation or no radiation. Log-rank test and/or pair wised comparison was used for significance. (**C** and **D**) Forest plots illustrate hazard ratios of subgroup univariate Cox regression of overall survival (**C**) and disease-free survival (**D**). Macrophages-Hi: Macrophages-High, Macrophages-Lo: Macrophages-Low, RT: radiation. This figure corresponds to the findings of our cohort as shown in **Figure 4**.


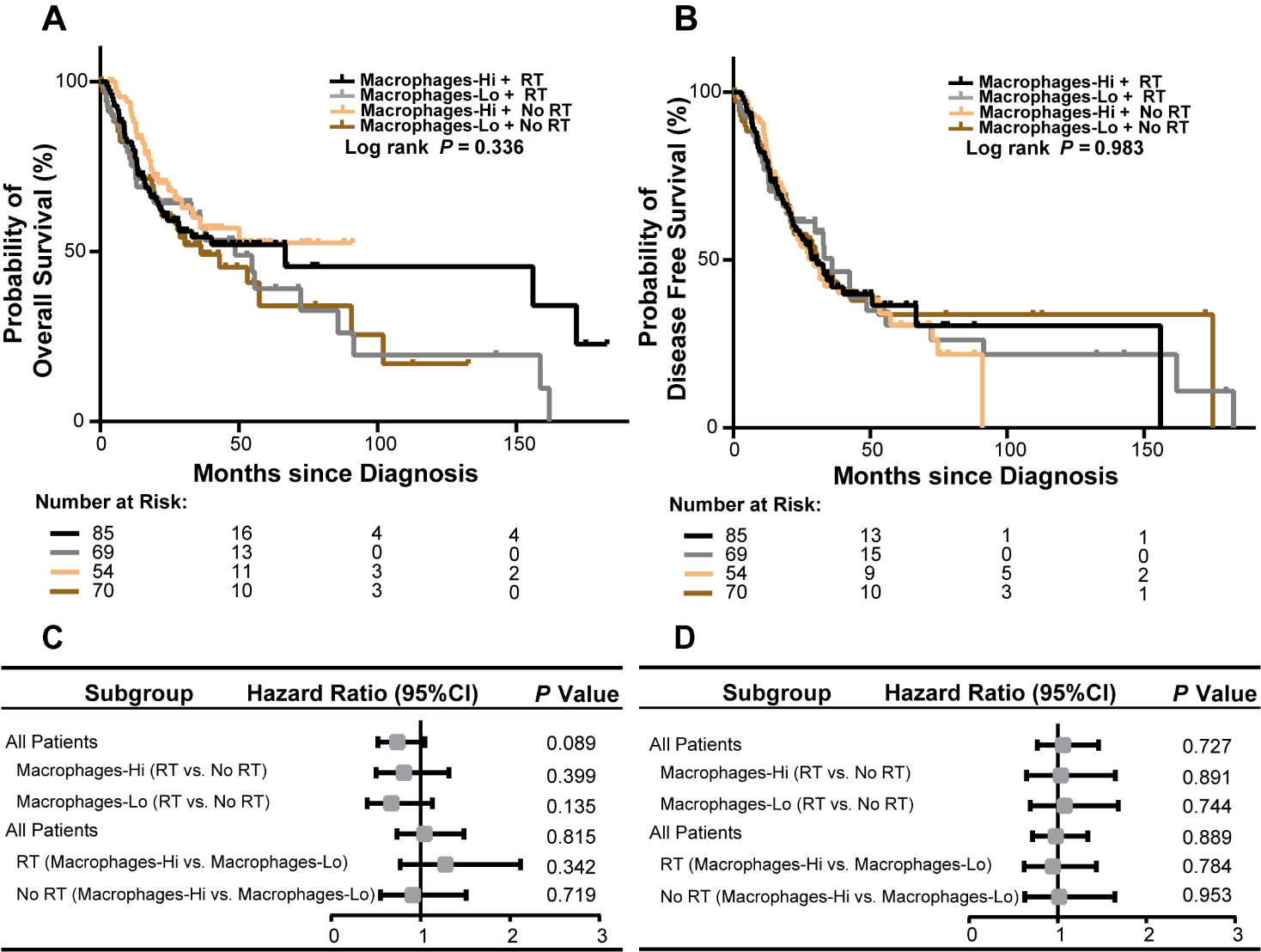


**Figure S6.** Association of macrophage infiltration deconvolved using EPIC and radiation with survival of all OSCC patients in TCGA cohort. (**A** and **B**) Kaplan-Meier curves show overall survival (**A**) and disease disease-free survival (**B**) in high or low macrophage infiltrated OSCC patients receiving radiation or no radiation. Log-rank test and/or pair wised comparison was used for significance. (**C** and **D**) Forest plots illustrate hazard ratios of subgroup univariate Cox regression of overall survival (**C**) and disease-free survival (**D**). Macrophages-Hi: Macrophages-High, Macrophages-Lo: Macrophages-Low, RT: radiation. This figure corresponds to the findings of our cohort as shown in **Figure 4**.

**
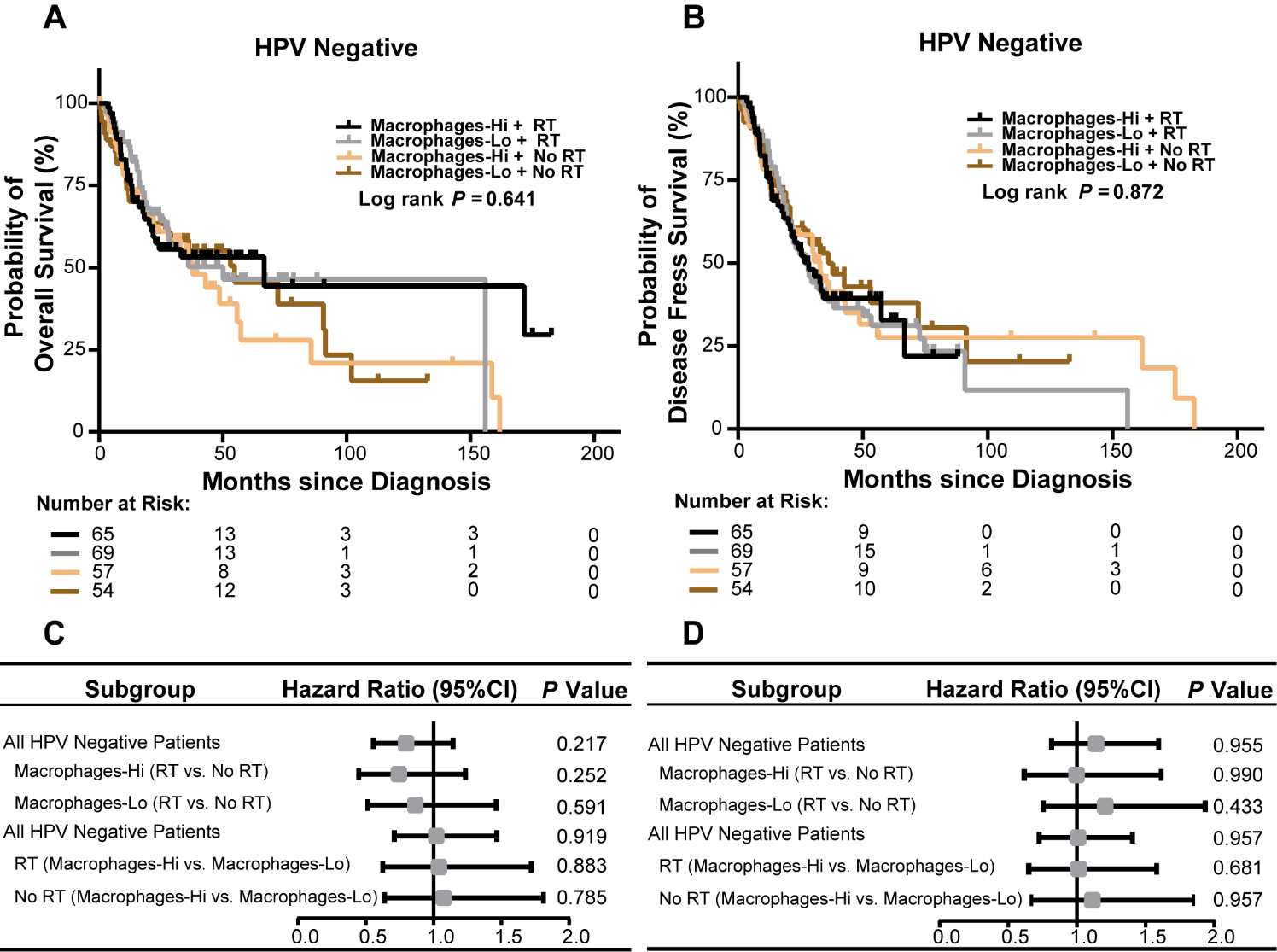
**

**Figure S7.** Association of macrophage infiltration deconvolved using CIBERSOFT and radiation with survival of HPV negative OSCC patients in TCGA cohort. (**A** and **B**) Kaplan-Meier curves exhibit overall survival (**A**) and disease-free survival (**B**) in high or low macrophage infiltrated HPV negative OSCC patients receiving radiation or no radiation. Log-rank test and/or pair wised comparison was used for significance. (**C** and **D**) Forest plots illustrate hazard ratios of subgroup univariate Cox regression of overall survival (**C**) and disease-free survival (**D**). Macrophages-Hi: Macrophages-High, Macrophages-Lo: Macrophages-Low, RT: radiation. This figure corresponds to the findings of our cohort as shown in **Figure 5**.


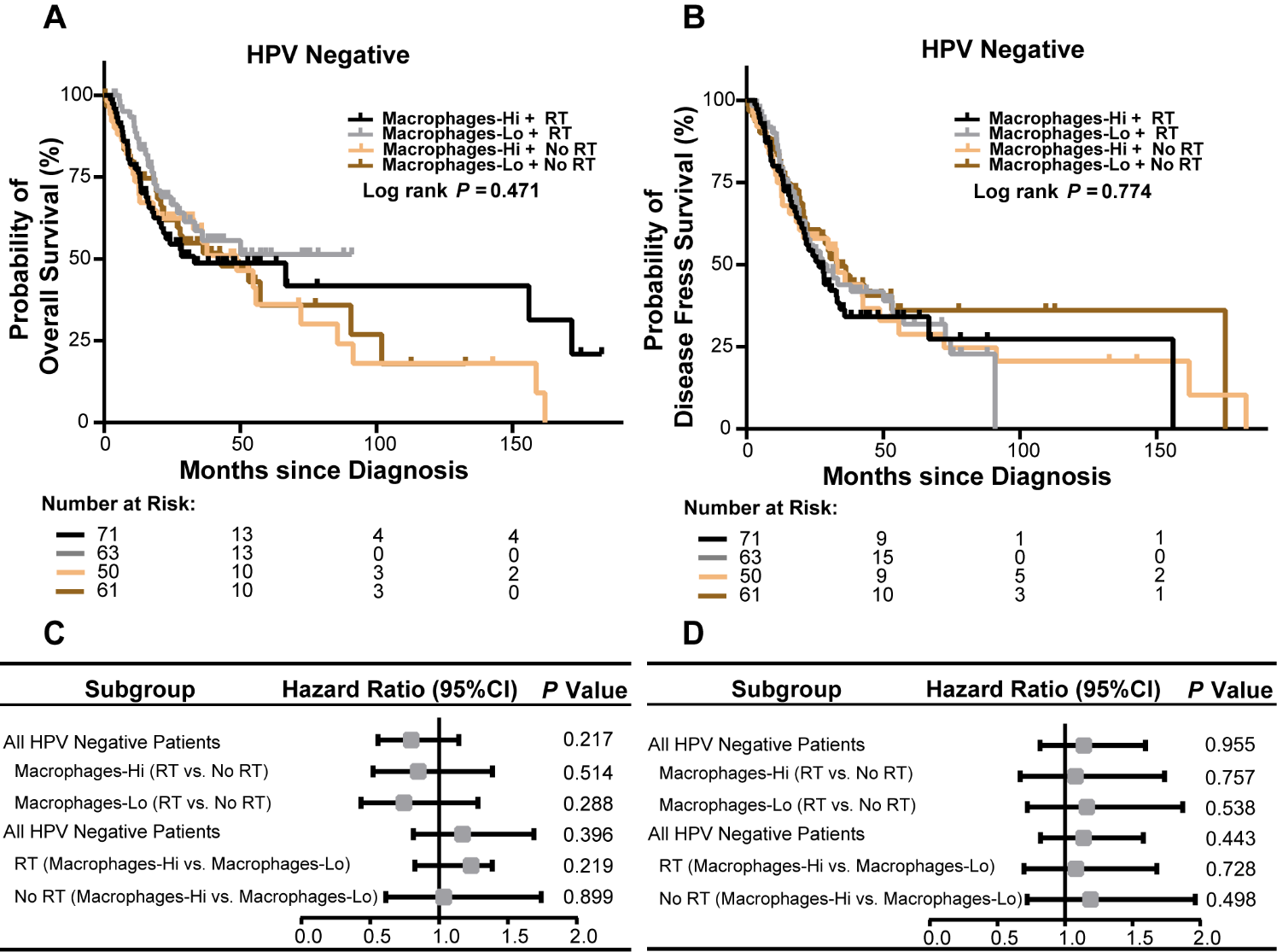


**Figure S8.** Association of macrophage infiltration deconvolved using EPIC and radiation with survival of HPV negative OSCC patients in TCGA cohort. (**A** and **B**) Kaplan-Meier curves exhibit overall survival (**A**) and disease-free survival (**B**) in high or low macrophage infiltrated HPV negative OSCC patients receiving radiation or no radiation. Log-rank test and/or pair wised comparison was used for significance. (**C** and **D**) Forest plots illustrate hazard ratios of subgroup univariate Cox regression of overall survival (**C**) and disease-free survival (**D**). Macrophages-Hi: Macrophages-High, Macrophages-Lo: Macrophages-Low, RT: radiation. This figure corresponds to the findings of our cohort as shown in **Figure 5**.

## Supplementary tables

**Table S1.** Baseline clinic-pathological characteristics of our cohort

| **Clinic-pathological**  **Characteristics** | **All Patients** | **HPV Negative** | **HPV Positive** |  | **CD68-High** | **CD68-Low** |  |
| --- | --- | --- | --- | --- | --- | --- | --- |
|  | **99 (100%)** | **74 (74.7%)** | **25 (25.3%)** | ***P*^b^** | **47 (47.5%)** | **52 (52.5%)** | ***P*^b^** |
|  | ***N (%)*** | ***N* (%)** | ***N* (%)** | **Value** | ***N (%)*** | ***N (%)*** | **Value** |
| Age |  |  |  | 0.326 |  |  | 0.054 |
| ≤ 59Y | 51 (51.5) | 36 (48.6) | 15 (60.0) |  | 29 (61.7) | 22 (42.3) |  |
| > 59Y | 48 (48.5) | 38 (51.4) | 10 (40.0) |  | 18 (38.3) | 30 (57.7) |  |
| Gender |  |  |  | 0.689 |  |  | 0.286 |
| Male | 56 (56.6) | 41 (55.4) | 15 (60.0) |  | 32 (68.1) | 24 (46.2) |  |
| Female | 43 (43.4) | 33 (44.6) | 10 (40.0) |  | 15 (31.9) | 28 (53.8) |  |
| Smoking Status |  |  |  | 0.439 |  |  | 0.530 |
| Smoking | 41 (41.4) | 29 (39.2) | 12 (48.0) |  | 21 (51.1) | 20 (32.7) |  |
| Non-smoking | 58 (58.6) | 45 (60.8) | 13 (52.0) |  | 26 (48.9) | 32 (67.3) |  |
| Drinking Status |  |  |  | 0.662 |  |  | 0.102 |
| Drinking | 36 (36.4) | 26 (35.1) | 10 (40.0) |  | 21 (44.7) | 15 (28.8) |  |
| Non-drinking | 63 (63.6) | 48 (64.9) | 15 (60.0) |  | 26 (55.3) | 37 (71.2) |  |
| Tumor Size |  |  |  | 0.384 |  |  | 0.625 |
| ≤ 2cm | 48 (48.5) | 34 (45.9) | 14 (56.0) |  | 24 (51.1) | 24 (46.2) |  |
| > 2cm | 51 (51.5) | 40 (54.1) | 11 (44.0) |  | 23 (48.9) | 28 (53.8) |  |
| Lymph Node |  |  |  | 0.600 |  |  | 0.196 |
| Positive | 36 (36.4) | 28 (37.8) | 8 (32.0) |  | 14 (29.8) | 22 (42.3) |  |
| Negative | 63 (63.6) | 46 (62.2) | 17 (68.0) |  | 33 (70.2) | 30 (57.7) |  |
| TNM Stage |  |  |  | 0.959 |  |  | 0.444 |
| Stage I-II | 55 (55.6) | 41 (55.4) | 14 (56.0) |  | 28 (59.6) | 27 (51.9) |  |
| Stage III-IV | 44 (44.4) | 33 (44.6) | 11 (44.0) |  | 19 (40.4) | 25 (48.1) |  |
| Differentiation |  |  |  | 0.882^a^ |  |  | 0.422 |
| High | 65 (65.7) | 48 (64.9) | 17 (68.0) |  | 28 (59.6) | 37 (71.2) |  |
| Moderate | 22 (22.2) | 16 (21.6) | 6 (24.0) |  | 13 (27.7) | 9 (17.3) |  |
| Poor | 12 (12.1) | 10 (13.5) | 2 (8.0) |  | 6 (12.7) | 6 (11.5) |  |
| Radiotherapy |  |  |  | 0.777 |  |  | 0.702 |
| Yes | 42 (42.4) | 32 (43.2) | 10 (40.0) |  | 19 (40.4) | 23 (44.2) |  |
| No | 57 (57.6) | 42 (56.8) | 15 (60.0) |  | 28 (59.6) | 29 (55.8) |  |

a: Fisher’s exact test. b: χ2 test except for those marked with a.

**Table S2.** Baseline clinic-pathological characteristics of TCGA cohort

| **Clinic-pathological**  **Characteristics** | **All Patients** | **HPV Negative** | **HPV Positive** |  |
| --- | --- | --- | --- | --- |
|  | **278 (100%)** | **245 (88.1%)** | **33 (11.9%)** |  |
|  | ***N (%)*** | ***N* (%)** | ***N* (%)** | ***P*^b^ Value** |
| Age |  |  |  | 0.426 |
| ≤ 61Y | 142 (51.1) | 123 (50.2) | 19 (57.6) |  |
| > 61Y | 136 (48.9) | 122 (49.8) | 14 (42.4) |  |
| Gender |  |  |  | 0.063 |
| Male | 188 (67.6) | 161 (65.7) | 27 (81.8) |  |
| Female | 90 (32.4) | 84 (32.3) | 6 (18.2) |  |
| Lymph Node |  |  |  | 0.569 |
| Positive | 143 (51.4) | 125 (51.0) | 18 (54.5) | 0.995***** |
| Negative | 111 (39.9) | 97 (39.6) | 14 (42.4) |  |
| NA | 24 (8.7) | 23 (9.4) | 1 (3.1) |  |
| TNM Stage |  |  |  | ＜0.001 |
| Stage I-II | 73 (26.3) | 62 (25.3) | 11 (33.3) | 0.001* |
| Stage III-IV | 198 (71.2) | 181 (73.9) | 17 (51.5) |  |
| NA | 7 (2.5) | 2 (0.8) | 5 (15.2) |  |
| Histological Grade | |  |  | 0.557 |
| G1-G2 | 218 (78.4) | 190 (77.6) | 28 (84.8) | 0.358* |
| G3-G4 | 59 (21.2) | 54 (22.0) | 5 (15.2) |  |
| NA | 1 (0.4) | 1 (0.4) | 0 (0.0) |  |
| Radiotherapy |  |  |  | 0.521 |
| Yes | 154 (55.4) | 134 (54.7) | 20 (60.6) |  |
| No | 124 (44.6) | 111 (45.3) | 13 (39.4) |  |

b: χ2 test. *Pairwise comparison without NA group.

**Table S3.** Univariate and multivariate cox regressions of overall survival of our cohort

|  | **Univariate** | |  | **Multivariate** | |
| --- | --- | --- | --- | --- | --- |
| **Variable** | **HR (95% CI)** | ***P* Value** |  | **HR (95% CI)** | ***P* Value** |
| CD68 (High vs. Low) | 0.881 (0.465-1.670) | 0.698 |  | 1.421 (0.557-2.258) | 0.704 |
| HPV Status (Positive vs. Negative) | 0.439 (0.183-1.053) | 0.065 |  | 0.448 (0.181-1.108) | 0.082 |
| Age (> 58 vs. ≤ 58) | 1.909 (0.994-3.667) | 0.052 |  | 1.750 (0.860-3.561) | 0.123 |
| Gender (Male vs. Female) | 1.529 (0.782-2.989) | 0.215 |  | 1.338 (0.640-2.797) | 0.439 |
| Smoking Status (Yes vs. No) | 1.086 (0.573-2.058) | 0.801 |  |  |  |
| Drinking Status (Yes vs. No) | 0.832 (0.426-1.628) | 0.592 |  |  |  |
| Tumor Size (> 2cm vs. ≤ 2cm) | 2.044 (1.056-3.955) | 0.034 |  | 1.544 (0.762-3.128) | 0.228 |
| Lymph Node (Positive vs. Negative) | 3.540 (1.843-6.800) | < 0.001 |  | 3.590 (1.818-7.089) | < 0.001 |
| TNM Stage (III-IV vs. I-II) | 3.454 (1.740-6.855) | < 0.001 |  |  |  |
| Differentiation |  |  |  |  |  |
| High | reference |  |  |  |  |
| Moderate | 0.949 (0.407-2.212) | 0.903 |  | 1.044 (0.434-2.510) | 0.923 |
| Poor | 2.652 (1.183-5.945) | 0.018 |  | 1.396 (0.575-3.391) | 0.461 |
| Radiotherapy (Yes vs. No) | 1.675 (0.885-3.168) | 0.113 |  |  |  |

**Table S4.** Univariate and multivariate cox regressions of disease-free survival of our cohort

|  | | **Univariate** | |  | **Multivariate** | |
| --- | --- | --- | --- | --- | --- | --- |
| **Variable** | | **HR (95% CI)** | ***P* Value** |  | **HR (95% CI)** | ***P* Value** |
| CD68 (High vs. Low) | 0.921 (0.522-1.625) | | 0.776 |  | 1.058 (0.580-1.932) | 0.854 |
| HPV Status (Positive vs. Negative) | 0.536 (0.259-1.110) | | 0.093 |  | 0.488 (0.228-1.041) | 0.063 |
| Age (> 58 vs. ≤ 58) | 1.277 (0.723-1.254) | | 0.399 |  | 1.109 (0.592-2.080) | 0.746 |
| Gender (Male vs. Female) | 1.645 (0.902-3.000) | | 0.104 |  | 1.654 (0.864-3.175) | 0.129 |
| Smoking Status (Yes vs. No) | 1.281 (0.727-2.258) | | 0.392 |  |  |  |
| Drinking Status (Yes vs. No) | 0.914 (0.513-1.631) | | 0.762 |  |  |  |
| Tumor Size (> 2cm vs. ≤ 2cm) | 1.549 (0.872-1.752) | | 0.135 |  | 0.324 (0.723-2.426) | 0.363 |
| Lymph Node (Positive vs. Negative) | 2.642 (1.493-4.676) | | 0.001 |  | 2.562 (1.410-4.653) | 0.002 |
| TNM Stage (III-IV vs. I-II) | 2.512 (1.405-4.494) | | 0.002 |  |  |  |
| Differentiation |  | |  |  |  |  |
| High | reference | |  |  |  |  |
| Moderate | 0.959 (0.469-1.963) | | 0.909 |  | 0.909 (0.434-2.906) | 0.802 |
| Poor | 1.799 (0.823-3.932) | | 0.141 |  | 1.067 (0.460-2.473) | 0.880 |
| Radiotherapy (Yes vs. No) | 1.148 (0.805-2.498) | | 0.227 |  |  |  |

**Table S5.** Univariate and multivariate cox regressions of overall survival of TCGA cohort

|  | **Univariate** | |  | **Multivariate^1^** | |  | **Multivariate^2^** | |
| --- | --- | --- | --- | --- | --- | --- | --- | --- |
| **Variable** | **HR (95% CI)** | ***P* Value** |  | **HR (95% CI)** | ***P* Value** |  | **HR (95% CI)** | ***P* Value** |
| Macrophages^1^ (High vs. Low) | 0.936 (0.659-1.328) | 0.710 | 0.952 (0.650-1.392) | | 0.798 |  |  |  |
| Macrophages^2^ (High vs. Low) | 1.043 (0.734-1.483) | 0.815 |  |  |  |  | 1.000 (0.682-1.466) | 1.000 |
| HPV Status (Positive vs. Negative) | 0.674 (0.328-1.387) | 0.284 | 0.605 (0.291-1.257) | | 0.178 |  | 0.600 (0.289-1.249) | 0.172 |
| Age (> 61Y vs. ≤ 61Y) | 1.135 (0.782-1.649) | 0.505 | 1.150 (0.767-1.725) | | 0.497 |  | 1.147 (0.766-1.719) | 0.505 |
| Gender (Male vs. Female) | 0.914 (0.615-1.359) | 0.657 | 0.997 (0.650-1.528) | | 0.989 |  | 0.996 (0.649-1.527) | 0.984 |
| Lymph Node (Positive vs. Negative) | 1.716 (1.157-2.545) | 0.007 | 1.734 (1.161-2.580) | | 0.007 |  | 1.737 (1.166-2.587) | 0.007 |
| TNM Stage (III-IV vs. I-II) | 1.694 (1.032-2.781) | 0.037 |  |  |  |  |  |  |
| Histological Grade (G3-G4 vs. G1-G2) | 1.277 (0.723-1.254) | 0.079 | 1.383 (0.897-2.133) | | 0.142 |  | 1.377 (0.894-2.122) | 0.146 |
| Radiotherapy (Yes vs. No) | 0.740 (0.523-1.047) | 0.089 |  |  |  |  |  |  |

^1^ Univariate and multivariate cox regressions of macrophage infiltration deconvolved using CIBERSOFT. ^2^ Univariate and multivariate cox regressions of macrophage infiltration deconvolved using EPIC.

**Table S6.** Univariate and multivariate cox regressions of disease-free survival of TCGA cohort

|  | **Univariate** | |  | | | **Multivariate^1^** | | |  | | **Multivariate^2^** | |
| --- | --- | --- | --- | --- | --- | --- | --- | --- | --- | --- | --- | --- |
| **Variable** | **HR (95% CI)** | ***P* Value** | |  | **HR (95% CI)** | | ***P* Value** |  | | **HR (95% CI)** | | ***P* Value** |
| Macrophages^1^ (High vs. Low) | 0.952 (0.694-1.305) | 0.758 | | 1.015 (0.723-1.425) | | | 0.931 |  | |  | |  |
| Macrophages^2^ (High vs. Low) | 0.978 (0.715-1.338) | 0.889 | |  |  | |  |  | | 0.961 (0.684-1.350) | | 0.819 |
| HPV Status (Positive vs. Negative) | 0.623 (0.327-1.188) | 0.151 | | 0.699 (0.390-1.253) | | | 0.229 |  | | 0.707 (0.394-1.270) | | 0.246 |
| Age (> 61Y vs. ≤ 61Y) | 1.275 (0.909-1.788) | 0.159 | | 1.389 (0.970-1.985) | | | 0.073 |  | | 1.391 (0.972-1.990) | | 0.071 |
| Gender (Male vs. Female) | 0.985 (0.686-1.414) | 0.935 | | 1.146 (0.783-1.679) | | | 0.484 |  | | 1.143 (0.780-1.675) | | 0.494 |
| Lymph Node (Positive vs. Negative) | 1.585 (1.116-2.249) | 0.010 | | 1.668 (1.175-2.369) | | | 0.004 |  | | 1.672 (1.177-2.376) | | 0.004 |
| TNM Stage (III-IV vs. I-II) | 1.985 (1.255-3.152) | 0.003 | |  |  | |  |  | |  | |  |
| Histological Grade (G3-G4 vs. G1-G2) | 1.464 (0.956-2.242) | 0.681 | | 1.002 (0.663-1.517) | | | 0.990 |  | | 1.006 (0.665-1.521) | | 0.977 |
| Radiotherapy (Yes vs. No) | 1.059 (0.768-1.459) | 0.727 | |  |  | |  |  | |  | |  |

^1^ Univariate and multivariate cox regressions of macrophage infiltration deconvolved using CIBERSOFT. ^2^ Univariate and multivariate cox regressions of macrophage infiltration deconvolved using EPIC.

**Table S7.** Subgroup cox regression of overall survival of OSCC patients in our cohort

|  |  | **Univariate** | |  | **Multivariate** | |
| --- | --- | --- | --- | --- | --- | --- |
| **Subgroup** | **Number** | **HR (95% CI)** | ***P* Value** |  | **HR (95% CI)** | ***P^#^* Value** |
| All Patients (RT vs. No RT) | 99 (42 vs. 57) | 1.675 (0.885-3.168) | 0.113 |  | 1.081 (0.534-2.188) | 0.829 |
| CD68-Hi (RT vs. No RT) | 47 (19 vs. 28) | 3.492 (1.287-9.476) | 0.014 |  | 1.339 (0.403-4.448) | 0.634 |
| CD68-Lo (RT vs. No RT) | 52 (23 vs. 29) | 0.922 (0.388-2.190) | 0.854 |  | 0.790 (0.290-2.091) | 0.635 |
| All Patients (CD68-Hi vs. CD68-Lo) | 99 (47 vs. 52) | 0.881 (0.465-1.670) | 0.698 |  | 1.142 (0.577-2.258) | 0.047 |
| RT (CD68-Hi vs. CD68-Lo) | 42 (19 vs. 23) | 1.753 (0.724-4.243) | 0.213 |  | 1.607 (0.581-4.443) | 0.361 |
| No RT (CD68-Hi vs. CD68-Lo) | 57 (28 vs. 29) | 0.449 (0.168-1.197) | 0.109 |  | 0.708 (0.243-2.060) | 0.526 |

^#^*P* values are adjusted by HPV status, age, gender, tumor size, lymph node metastasis and differentiation.

**Table S8.** Subgroup univariate cox regression of disease-free survival of OSCC patients in our cohort

|  |  | **Univariate** | |  | **Multivariate** | |
| --- | --- | --- | --- | --- | --- | --- |
| **Subgroup** | **Number** | **HR (95% CI)** | ***P* Value** |  | **HR (95% CI)** | ***P^#^* Value** |
| All Patients (RT vs. No RT) | 99 (42 vs. 57) | 1.148 (0.805-2.498) | 0.227 |  | 0.890 (0.470-1.686) | 0.721 |
| CD68-Hi (RT vs. No RT) | 47 (19 vs. 28) | 2.610 (1.112-6.123) | 0.027 |  | 1.025 (0.358-2.940) | 0.963 |
| CD68-Lo (RT vs. No RT) | 52 (23 vs. 29) | 0.845 (0.388-1.842) | 0.672 |  | 0.602 (0.253-1.433) | 0.252 |
| All Patients (CD68-Hi vs. CD68-Lo) | 99 (47 vs. 52) | 0.921 (0.522-1.625) | 0.776 |  | 1.058 (0.580-1.932) | 0.854 |
| RT (CD68-Hi vs. CD68-Lo) | 42 (19 vs. 23) | 1.700 (0.760-3.800) | 0.190 |  | 1.490 (0.602-3.687) | 0.388 |
| No RT (CD68-Hi vs. CD68-Lo) | 57 (28 vs. 29) | 0.543 (0.237-1.241) | 0.148 |  | 0.664 (0.260-1.698) | 0.393 |

^#^*P* values are adjusted by HPV status, age, gender, tumor size, lymph node metastasis and differentiation.

**Table S9.** Subgroup cox regression of overall survival of HPV negative OSCC patients in our cohort

|  |  | **Univariate** |  | | **Multivariate** | |
| --- | --- | --- | --- | --- | --- | --- |
| **Subgroup** | **Number** | **HR (95% CI)** | ***P* Value** |  | **HR (95% CI)** | ***P^#^* Value** |
| All HPV Negative Patients (RT vs. No RT) | 74 (32 vs. 42) | 1.858 (0.924-3.739) | 0.082 |  | 1.314 (0.611-2.829) | 0.485 |
| CD68-Hi (RT vs. No RT) | 33 (13 vs. 20) | 3.746 (1.248-11.244) | 0.019 |  | 1.117 (0.299-4.175) | 0.869 |
| CD68-Lo (RT vs. No RT) | 41 (19 vs. 22) | 1.133 (0.449-2.855) | 0.791 |  | 1.341 (0.463-3.885) | 0.589 |
| All HPV Negative Patients (CD68-Hi vs. CD68-Lo) | 74 (33 vs. 41) | 1.000 (0.520-1.992) | 1.000 |  | 1.000 (0.520-1.992) | 1.000 |
| RT (CD68-Hi vs. CD68-Lo) | 32 (13 vs. 19) | 1.922 (0.751-4.916) | 0.173 |  | 1.114 (0.353-3.522) | 0.854 |
| No RT (CD68-Hi vs. CD68-Lo) | 42 (20 vs. 22) | 0.540 (0.181-1.612) | 0.270 |  | 1.180 (0.296-4.707) | 0.815 |

^#^*P* values are adjusted by age, gender, tumor size, lymph node metastasis and differentiation.

**Table S10.** Subgroup cox regression of disease-free survival of HPV negative OSCC patients in our cohort

|  |  | **Univariate** | |  | **Multivariate** | |
| --- | --- | --- | --- | --- | --- | --- |
| **Subgroup** | **Number** | **HR (95% CI)** | ***P* Value** |  | **HR (95% CI)** | ***P^#^* Value** |
| All HPV Negative Patients (RT vs. No RT) | 74 (32 vs. 42) | 1.525 (0.812-2.865) | 0.189 |  | 1.067 (0.523-2.178) | 0.859 |
| CD68-Hi (RT vs. No RT) | 33 (13 vs. 20) | 3.012 (1.161-7.814) | 0.023 |  | 1.037 (0.305-3.553) | 0.953 |
| CD68-Lo (RT vs. No RT) | 41 (19 vs. 22) | 0.914 (0.388-2.215) | 0.838 |  | 0.881 (0.335-2.320) | 0.798 |
| All HPV Negative Patients (CD68-Hi vs. CD68-Lo) | 74 (33 vs. 41) | 1.000 (0.546-1.831) | 1.000 |  | 1.000 (0.546-1.831) | 1.000 |
| RT (CD68-Hi vs. CD68-Lo) | 32 (13 vs. 19) | 2.105 (0.886-5.004) | 0.092 |  | 1.216 (0.633-2.338) | 0.859 |
| No RT (CD68-Hi vs. CD68-Lo) | 42 (20 vs. 22) | 0.628 (0.243-1.625) | 0.338 |  | 0.814 (0.220-3.007) | 0.758 |

^#^*P* values are adjusted by age, gender, tumor size, lymph node metastasis and differentiation.

**Table S11.** Subgroup cox regression of overall survival of HPV positive OSCC patients in our cohort

|  |  | **Univariate** | |  | **Multivariate** | |
| --- | --- | --- | --- | --- | --- | --- |
| **Subgroup** | **Number** | **HR (95% CI)** | ***P* Value** |  | **HR (95% CI)** | ***P^#^* Value** |
| All HPV Positive Patients (RT vs. No RT) | 25 (10 vs. 15) | 0.811 (0.148-4.434) | 0.809 |  | NA | NA |
| CD68-Hi (RT vs. No RT) | 14 (6 vs. 8) | 3.109 (0.281-34.406) | 0.355 |  | NA | NA |
| CD68-Lo (RT vs. No RT) | 11 (4 vs. 7) | NA | NA |  | NA | NA |
| All HPV Positive Patients (CD68-Hi vs. CD68-Lo) | 25 (14 vs. 11) | 0.807 (0.163-4.005) | 0.739 |  | 1.588 (0.185-13.644) | 0.674 |
| RT (CD68-Hi vs. CD68-Lo) | 10 (6 vs. 4) | NA | NA |  | NA | NA |
| No RT (CD68-Hi vs. CD68-Lo) | 15 (8 vs. 7) | 0.230 (0.024-2.227) | 0.205 |  | 0.131 (0.001-32.899) | 0.471 |

^#^*P* values are adjusted by age, gender, tumor size, lymph node metastasis and differentiation. NA: Cox regression is not applicable.

**Table S12.** Subgroup cox regression of disease-free survival of HPV positive OSCC patients in our cohort

|  |  | **Univariate** | |  | **Multivariate** | |
| --- | --- | --- | --- | --- | --- | --- |
| **Subgroup** | **Number** | **HR (95% CI)** | ***P* Value** |  | **HR (95% CI)** | ***P^#^* Value** |
| All HPV Positive Patients (RT vs. No RT) | 25 (10 vs. 15) | 0.834 (0.208-3.338) | 0.797 |  | 1.969 (0.172-22.539) | 0.586 |
| CD68-Hi (RT vs. No RT) | 14 (6 vs. 8) | 1.579 (0.221-11.296) | 0.649 |  | NA | NA |
| CD68-Lo (RT vs. No RT) | 11 (4 vs. 7) | 0.406 (0.045-3.647) | 0.421 |  | NA | NA |
| All HPV Positive Patients (CD68-Hi vs. CD68-Lo) | 25 (14 vs. 11) | 0.628 (0.168-2.344) | 0.489 |  | 0.642 (0.113-3.643) | 0.617 |
| RT (CD68-Hi vs. CD68-Lo) | 10 (6 vs. 4) | 1.535 (0.138-17.103) | 0.727 |  | NA | NA |
| No RT (CD68-Hi vs. CD68-Lo) | 15 (8 vs. 7) | 0.364 (0.066-1.999) | 0.245 |  | 0.885 (0.038-20.885) | 0.940 |

^#^*P* values are adjusted by age, gender, tumor size, lymph node metastasis and differentiation. NA: Cox regression is not applicable.

**Table S13.** Subgroup cox regression of overall survival of OSCC patients with macrophage infiltration deconvolved using CIBERSOFT in TCGA cohort

|  |  | **Univariate** | |  | **Multivariate** | |
| --- | --- | --- | --- | --- | --- | --- |
| **Subgroup** | **Number** | **HR (95% CI)** | ***P* Value** |  | **HR (95% CI)** | ***P^#^* Value** |
| All Patients (RT vs. No RT) | 278 (154 vs. 124) | 0.740 (0.522-1.047) | 0.089 |  | 0.689 (0.467-1.016) | 0.060 |
| Macrophages-Hi (RT vs. No RT) | 139 (77 vs. 62) | 0.666 (0.406-1.092) | 0.107 |  | 0.687 (0.386-1.220) | 0.200 |
| Macrophages-Lo (RT vs. No RT) | 139 (77 vs. 62) | 0.827 (0.503-1.360) | 0.454 |  | 0.738 (0.434-1.257) | 0.264 |
| All Patients (Macrophages-Hi vs. Macrophages-Lo) | 278 (139 vs. 139) | 0.936 (0.659-1.328) | 0.710 |  | 0.952 (0.650-1.392) | 0.798 |
| RT (Macrophages-Hi vs. Macrophages-Lo) | 154 (77 vs. 77) | 0.918 (0.561-1.501) | 0.732 |  | 0.981 (0.589-1.635) | 0.943 |
| No RT (Macrophages-Hi vs. Macrophages-Lo) | 124 (62 vs. 62) | 1.017 (0.618-1.675) | 0.947 |  | 1.125 (0.629-2.041) | 0.691 |

^#^*P* values are adjusted by HPV status, age, gender, lymph node metastasis and histological grade.

**Table S14.** Subgroup univariate cox regression of disease-free survival of OSCC patients with macrophage infiltration deconvolved using CIBERSOFT in TCGA cohort

|  |  | **Univariate** | |  | **Multivariate** | |
| --- | --- | --- | --- | --- | --- | --- |
| **Subgroup** | **Number** | **HR (95% CI)** | ***P* Value** |  | **HR (95% CI)** | ***P^#^* Value** |
| All Patients (RT vs. No RT) | 278 (154 vs. 124) | 1.059 (0.768-1.459) | 0.727 |  | 1.025 (0.722-1.456) | 0.888 |
| Macrophages-Hi (RT vs. No RT) | 139 (77 vs. 62) | 0.914 (0.576-1.450) | 0.702 |  | 0.932 (0.556-1.562) | 0.788 |
| Macrophages-Lo (RT vs. No RT) | 139 (77 vs. 62) | 1.137 (0.728-1.774) | 0.573 |  | 1.083 (0.673-1.742) | 0.744 |
| All Patients (Macrophages-Hi vs. Macrophages-Lo) | 278 (139 vs. 139) | 0.952 (0.694-1.305) | 0.758 |  | 1.015 (0.723-1.425) | 0.931 |
| RT (Macrophages-Hi vs. Macrophages-Lo) | 154 (77 vs. 77) | 0.930 (0.609-1.420) | 0.737 |  | 1.015 (0.653-1.577) | 0.948 |
| No RT (Macrophages-Hi vs. Macrophages-Lo) | 124 (62 vs. 62) | 1.064 (0.656-1.725) | 0.803 |  | 1.101 (0.640-1.891) | 0.729 |

^#^*P* values are adjusted by HPV status, age, gender, lymph node metastasis and histological grade.

**Table S15.** Subgroup cox regression of overall survival of OSCC patients with macrophage infiltration deconvolved using EPIC in TCGA cohort

|  |  | **Univariate** | |  | **Multivariate** | |
| --- | --- | --- | --- | --- | --- | --- |
| **Subgroup** | **Number** | **HR (95% CI)** | ***P* Value** |  | **HR (95% CI)** | ***P^#^* Value** |
| All Patients (RT vs. No RT) | 278 (154 vs. 124) | 0.740 (0.522-1.047) | 0.089 |  | 0.685 (0.464-1.012) | 0.058 |
| Macrophages-Hi (RT vs. No RT) | 139 (85 vs. 54) | 0.812 (0.502-1.316) | 0.399 |  | 0.935 (0.526-1.662) | 0.820 |
| Macrophages-Lo (RT vs. No RT) | 139 (69 vs. 70) | 0.673 (0.401-1.131) | 0.135 |  | 0.572 (0.328-1.197) | 0.149 |
| All Patients (Macrophages-Hi vs. Macrophages-Lo) | 278 (139 vs. 139) | 1.043 (0.734-1.483) | 0.815 |  | 1.000 (0.682-1.466) | 1.000 |
| RT (Macrophages-Hi vs. Macrophages-Lo) | 154 (85 vs. 69) | 1.277 (0.771-2.117) | 0.342 |  | 1.355 (0.801-2.306) | 0.255 |
| No RT (Macrophages-Hi vs. Macrophages-Lo) | 124 (54 vs. 70) | 0.912 (0.551-1.509) | 0.719 |  | 0.868 (0.484-1.558) | 0.635 |

^#^*P* values are adjusted by HPV status, age, gender, lymph node metastasis and histological grade.

**Table S16.** Subgroup univariate cox regression of disease-free survival of OSCC patients with macrophage infiltration deconvolved using EPIC in TCGA cohort

|  |  | **Univariate** | |  | **Multivariate** | |
| --- | --- | --- | --- | --- | --- | --- |
| **Subgroup** | **Number** | **HR (95% CI)** | ***P* Value** |  | **HR (95% CI)** | ***P^#^* Value** |
| All Patients (RT vs. No RT) | 278 (154 vs. 124) | 1.059 (0.768-1.459) | 0.727 |  | 1.031 (0.725-1.468) | 0.864 |
| Macrophages-Hi (RT vs. No RT) | 139 (85 vs. 54) | 1.033 (0.648-1.648) | 0.891 |  | 1.149 (0.669-1.973) | 0.614 |
| Macrophages-Lo (RT vs. No RT) | 139 (69 vs. 70) | 1.077 (0.689-1.683) | 0.744 |  | 1.034 (0.638-1.677) | 0.892 |
| All Patients (Macrophages-Hi vs. Macrophages-Lo) | 278 (139 vs. 139) | 0.978 (0.715-1.338) | 0.889 |  | 0.961 (0.684-1.350) | 0.819 |
| RT (Macrophages-Hi vs. Macrophages-Lo) | 154 (85 vs. 69) | 0.943 (0.620-1.435) | 0.784 |  | 1.009 (0.646-1.575) | 0.970 |
| No RT (Macrophages-Hi vs. Macrophages-Lo) | 124 (54 vs. 70) | 1.015 (0.627-1.643) | 0.953 |  | 0.942 (0.537-1.650) | 0.834 |

^#^*P* values are adjusted by HPV status, age, gender, lymph node metastasis and histological grade.

**Table S17.** Subgroup cox regression of overall survival of HPV negative OSCC patients with macrophage infiltration deconvolved using CIBERSOFT in TCGA cohort

|  |  | **Univariate** | | |  | | **Multivariate** | | |
| --- | --- | --- | --- | --- | --- | --- | --- | --- | --- |
| **Subgroup** | **Number** | **HR (95% CI)** | ***P* Value** | |  | **HR (95% CI)** | | ***P^#^* Value** | |
| All HPV Negative Patients (RT vs. No RT) | 245 (134 vs. 111) | 0.797 (0.556-1.143) | | 0.217 |  | 0.746 (0.498-1.118) | | | 0.155 |
| Macrophages-Hi (RT vs. No RT) | 122 (65 vs. 57) | 0.743 (0.448-1.235) | | 0.252 |  | 0.743 (0.412-1.340) | | | 0.323 |
| Macrophages-Lo (RT vs. No RT) | 123 (69 vs. 54) | 0.866 (0.514-1.462) | | 0.591 |  | 0.780 (0.445-1.368) | | | 0.385 |
| All HPV Negative Patients (Macrophages-Hi vs. Macrophages-Lo) | 245 (122 vs. 123) | 1.019 (0.709-1.466) | | 0.919 |  | 1.025 (0.690-1.522) | | | 0.902 |
| RT (Macrophages-Hi vs. Macrophages-Lo) | 134 (65 vs. 69) | 1.039 (0.625-1.720) | | 0.883 |  | 1.040 (0.616-1.754) | | | 0.884 |
| No RT (Macrophages-Hi vs. Macrophages-Lo) | 111 (57 vs. 54) | 1.075 (0.637-1.811) | | 0.785 |  | 1.169 (0.634-2.156) | | | 0.617 |

^#^*P* values are adjusted by age, gender, lymph node metastasis and histological grade.

**Table S18.** Subgroup cox regression of disease-free survival of HPV negative OSCC patients with macrophage infiltration deconvolved using CIBERSOFT in TCGA cohort

|  |  | **Univariate** | | |  | | **Multivariate** | | |
| --- | --- | --- | --- | --- | --- | --- | --- | --- | --- |
| **Subgroup** | **Number** | **HR (95% CI)** | ***P* Value** | |  | **HR (95% CI)** | | ***P^#^* Value** | |
| All HPV Negative Patients (RT vs. No RT) | 245 (134 vs. 111) | 1.140 (0.815-1.596) | | 0.955 |  | 1.105 (0.764-1.597) | | | 0.596 |
| Macrophages-Hi (RT vs. No RT) | 122 (65 vs. 57) | 0.997 (0.616-1.613) | | 0.990 |  | 0.959 (0.561-1.638) | | | 0.877 |
| Macrophages-Lo (RT vs. No RT) | 123 (69 vs. 54) | 1.207 (0.754-1.934) | | 0.433 |  | 1.182 (0.713-1.961) | | | 0.516 |
| All HPV Negative Patients (Macrophages-Hi vs. Macrophages-Lo) | 245 (122 vs. 123) | 1.009 (0.725-1.405) | | 0.957 |  | 1.080 (0.757-1.540) | | | 0.673 |
| RT (Macrophages-Hi vs. Macrophages-Lo) | 134 (65 vs. 69) | 1.013 (0.649-1.581) | | 0.681 |  | 1.055 (0.666-1.670) | | | 0.821 |
| No RT (Macrophages-Hi vs. Macrophages-Lo) | 111 (57 vs. 54) | 1.112 (0.669-1.848) | | 0.957 |  | 1.158 (0.654-1.049) | | | 0.616 |

^#^*P* values are adjusted by age, gender, lymph node metastasis and histological grade.

**Table S19.** Subgroup cox regression of overall survival of HPV negative OSCC patients with macrophage infiltration deconvolved using EPIC in TCGA cohort

|  |  | **Univariate** | | |  | | **Multivariate** | | |
| --- | --- | --- | --- | --- | --- | --- | --- | --- | --- |
| **Subgroup** | **Number** | **HR (95% CI)** | ***P* Value** | |  | **HR (95% CI)** | | ***P^#^* Value** | |
| All HPV Negative Patients (RT vs. No RT) | 245 (134 vs. 111) | 0.797 (0.556-1.143) | | 0.217 |  | 0.742 (0.495-1.112) | | | 0.148 |
| Macrophages-Hi (RT vs. No RT) | 121 (71 vs. 50) | 0.849 (0.520-1.387) | | 0.514 |  | 0.905 (0.505-1.620) | | | 0.736 |
| Macrophages-Lo (RT vs. No RT) | 124 (63 vs. 61) | 0.745 (0.433-1.282) | | 0.288 |  | 0.670 (0.376-1.193) | | | 0.174 |
| All HPV Negative Patients (Macrophages-Hi vs. Macrophages-Lo) | 245 (121 vs. 124) | 1.171 (0.813-1.689) | | 0.396 |  | 1.101 (0.739-1.639) | | | 0.636 |
| RT (Macrophages-Hi vs. Macrophages-Lo) | 134 (71 vs. 63) | 1.386 (0.824-1.233) | | 0.219 |  | 1.301 (0.761-2.226) | | | 0.336 |
| No RT (Macrophages-Hi vs. Macrophages-Lo) | 111 (111 vs. 50) | 1.034 (0.614-1.741) | | 0.899 |  | 1.014 (0.551-1.864) | | | 0.965 |

^#^*P* values are adjusted by age, gender, lymph node metastasis and histological grade.

**Table S20.** Subgroup cox regression of disease-free survival of HPV negative OSCC patients with macrophage infiltration deconvolved using EPIC in TCGA cohort

|  |  | **Univariate** | | |  | | **Multivariate** | | |
| --- | --- | --- | --- | --- | --- | --- | --- | --- | --- |
| **Subgroup** | **Number** | **HR (95% CI)** | ***P* Value** | |  | **HR (95% CI)** | | ***P^#^* Value** | |
| All HPV Negative Patients (RT vs. No RT) | 245 (134 vs. 111) | 1.140 (0.815-1.596) | | 0.955 |  | 1.097 (0.758-1.588) | | | 0.623 |
| Macrophages-Hi (RT vs. No RT) | 121 (71 vs. 50) | 1.078 (0.669-1.737) | | 0.757 |  | 1.079 (0.625-1.865) | | | 0.784 |
| Macrophages-Lo (RT vs. No RT) | 124 (63 vs. 61) | 1.162 (0.721-1.871) | | 0.538 |  | 1.146 (0.685-1.917) | | | 0.603 |
| All HPV Negative Patients (Macrophages-Hi vs. Macrophages-Lo) | 245 (121 vs. 124) | 1.137 (0.819-1.58) | | 0.443 |  | 1.082 (0.756-1.547) | | | 0.667 |
| RT (Macrophages-Hi vs. Macrophages-Lo) | 134 (71 vs. 63) | 1.081 (0.696-1.68) | | 0.728 |  | 1.062 (0.671-1.683) | | | 0.797 |
| No RT (Macrophages-Hi vs. Macrophages-Lo) | 111 (111 vs. 50) | 1.189 (0.72-1.964) | | 0.498 |  | 1.114 (0.618-2.007) | | | 0.719 |

^#^*P* values are adjusted by age, gender, lymph node metastasis and histological grade.
